# PDZK1 suppresses TNBC development and sensitizes TNBC cells to erlotinib via the EGFR pathway

**DOI:** 10.1038/s41419-024-06502-2

**Published:** 2024-04-12

**Authors:** Yuanzhen Ma, Zhiyu Fang, Hongning Zhang, Yijun Qi, Yuke Mao, Junfang Zheng

**Affiliations:** 1 Beijing Key Laboratory of Cancer Invasion and Metastasis Research, Department of Biochemistry and Molecular Biology, School of Basic Medical Sciences, Capital Medical University, 100069 Beijing, China; 2 Department of Pharmacology, School of Basic Medical Sciences, Capital Medical University, 100069 Beijing, China

**Keywords:** Breast cancer, Growth factor signalling, Oncogenesis, Tumour-suppressor proteins, Targeted therapies

## Abstract

Epidermal growth factor receptor (EGFR)-targeted drugs (erlotinib, etc.) are used to treat multiple types of tumours. EGFR is highly expressed in most triple-negative breast cancer (TNBC) patients. However, only a small proportion of TNBC patients benefit from EGFR-targeted drugs in clinical trials, and the resistance mechanism is unclear. Here, we found that PDZ domain containing 1 (PDZK1) is downregulated in erlotinib-resistant TNBC cells, suggesting that PDZK1 downregulation is related to erlotinib resistance in TNBC. PDZK1 binds to EGFR. Through this interaction, PDZK1 promotes EGFR degradation by enhancing the binding of EGFR to c-Cbl and inhibits EGFR phosphorylation by hindering EGFR dimerisation. We also found that PDZK1 is specifically downregulated in TNBC tissues and correlated with a poor prognosis in TNBC patients. In vitro and in vivo functional assays showed that PDZK1 suppressed TNBC development. Restoration of EGFR expression or kinase inhibitor treatment reversed the degree of cell malignancy induced by PDZK1 overexpression or knockdown, respectively. PDZK1 overexpression sensitised TNBC cells to erlotinib both in vitro and in vivo. In conclusion, PDZK1 is a significant prognostic factor for TNBC and a potential molecular therapeutic target for reversing erlotinib resistance in TNBC cells.

## Introduction

Among breast cancer subtypes, triple-negative breast cancer (TNBC) has the worst prognosis and the lowest metastasis-free survival rate. Currently, there is no effective targeted therapy for TNBC. One of the characteristics that distinguishes TNBC from other breast cancer types is that most (approximately 50–80%) TNBC patients highly express epidermal growth factor receptor (EGFR) [1, 2]. High EGFR expression predicts a poor prognosis in TNBC patients [3]. EGFR is a transmembrane receptor tyrosine kinase. EGFR is often activated in TNBC cells [1, 4–6]. EGFR-mediated activation of the PI3K/AKT or RAS/RAF/MEK/ERK pathways plays an important role in cell proliferation, migration and inhibition of apoptosis [7, 8]. Therefore, EGFR is a promising molecular target for TNBC treatment [9, 10]. The development of EGFR-targeted drugs and improvements in the effectiveness of EGFR-targeted drugs have attracted extensive interest [11].

EGFR monoclonal antibodies (mAbs, cetuximab, etc.) can degrade EGFR [12], and EGFR tyrosine kinase inhibitors (TKIs, erlotinib, gefitinib, etc.) can function by competitively binding to the ATP site within the catalytic domain and preventing the phosphorylation/activation of EGFR targets, including AKT and ERK. These types of EGFR-targeted drugs have been tested in clinical trials. However, the effect was not satisfactory [13–17]. Only 3% or 6% of TNBC patients in stage IV partially responded to combined erlotinib/bevacizumab therapy or cetuximab, respectively [15, 18], and 17% of early TNBC patients had a pathological complete response (pCR) after treatment with gefitinib combined with neoadjuvant chemotherapeutic drugs [16]. These findings suggested that most TNBC patients were resistant to EGFR-targeted drugs. The drawback of these clinical trials was that the EGFR level was not used to stratify TNBC patients who would benefit from EGFR-targeted therapy. In a subsequent clinical trial, TNBC patients with high EGFR levels and stage II/IIIa disease were prescreened for EGFR-targeted therapy. Nevertheless, the pCR rate did not increase to 100% [17]. In a grade 3 TNBC patient-derived tumour xenograft (PDX) model in nude mice, mice engrafted with TNBC cells with high EGFR levels exhibited differential responses to EGFR-targeted drugs [19]. These findings suggested that there might be an unknown mechanism leading to the activation of the EGFR signalling pathway and resistance to EGFR-targeted drugs in TNBC cells. Therefore, clarifying the detailed mechanism of EGFR signalling pathway activation in TNBC patients will help to elucidate the mechanism of TNBC resistance to EGFR-targeted drugs and will provide a new target for reversing resistance to EGFR-targeted drugs and a theoretical basis for precisely stratifying TNBC patients for treatment.

Excessive activation of the EGFR signalling pathway is usually due to functional abnormalities in regulatory proteins [20]. Regulatory proteins also play an important role in sensitising cells to EGFR-targeted drugs [21, 22]. PSD-95/Discs large/ZO-1 (PDZ) proteins are regulatory proteins. PDZ proteins negatively regulate tumour signalling pathways and inhibit tumour development by binding to tumour-related proteins containing PDZ protein binding motifs [23]. The carboxyl terminal (CT) of EGFR also contains the PDZ protein binding motif DSFL. The PDZ protein Na^+^/H^+^ exchanger regulatory factor (NHERF) binds to EGFR and inhibits the activation of the EGFR pathway [24, 25]. However, the NHERF level is not changed in the tissues of patients with advanced-stage TNBC [26]. Therefore, NHERF does not play a role in TNBC progression.

In this study, we discovered a new negative regulatory mechanism for EGFR signalling and a novel function of PDZ domain containing 1 (PDZK1) in TNBC. Abnormal downregulation of PDZK1 may constitute an essential mechanism underlying TNBC development and resistance to EGFR-targeted therapy, and the PDZK1-EGFR axis may represent a potential therapeutic target in TNBC.

## Results

### PDZK1 is correlated with TNBC development and erlotinib sensitivity

**Fig. 1:**
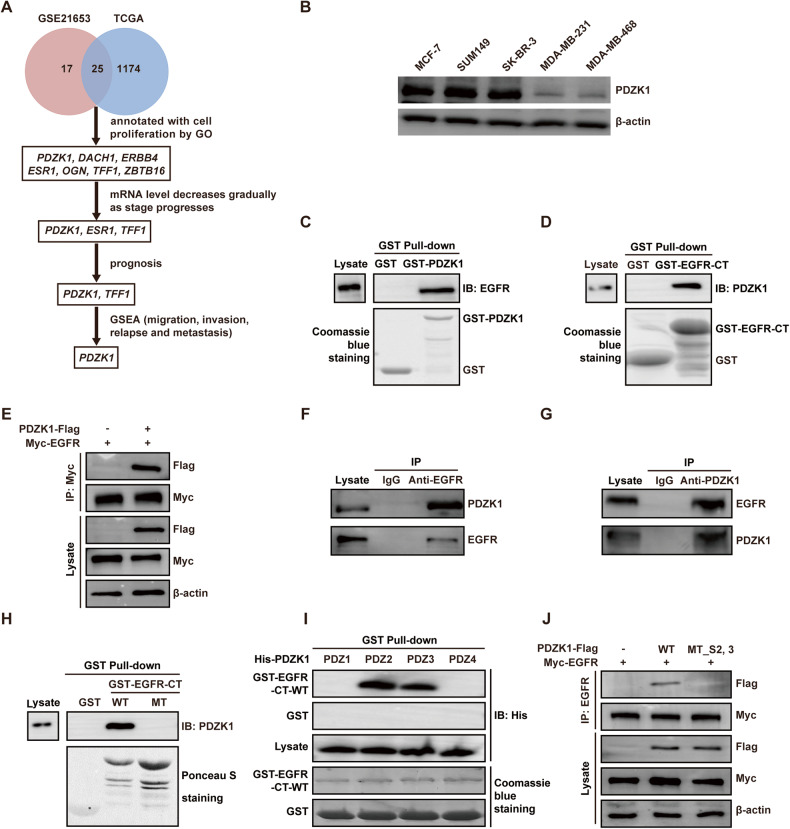
PDZK1 correlated with TNBC development and erlotinib sensitivity is a novel EGFR-binding protein. **A** The screening flowchart of critical genes in TNBC. The common genes differentially expressed both in GSE21653 gene chip data and TCGA_BRCA dataset were selected. Then, these genes were annotated with GO and their correlations with stage, prognosis, migration, invasion, relapse and metastasis were analysed. **B** PDZK1 protein level was downregulated in erlotinib-resistant TNBC cells. The PDZK1 protein levels in these cells were detected by WB. **C**, **D** EGFR was a novel binding protein of PDZK1. Purified Glutathione S-Transferase (GST) and GST-PDZK1 fusion proteins (**C**)/GST-EGFR-Carboxyl terminal (CT) fusion proteins (**D**) were used in pull-down assay with lysates of HEK293 cells. Precipitates were subjected to WB with the anti-EGFR antibody (**C**, top panel)/anti-PDZK1 antibody (**D**, top panel). Coomassie Blue staining demonstrated equal loading of the fusion proteins. **E** Exogenous Co-IP of PDZK1 with EGFR. MDA-MB-231 cells transfected with constructs of PDZK1-Flag and Myc-EGFR were solubilized and incubated with anti-Myc antibody coupled to affinity gel. Co-IP of PDZK1-Flag with Myc-EGFR was evident and it was not detectable when Myc-EGFR was expressed alone. **F**, **G** Endogenous Co-IP of PDZK1 with EGFR. MDA-MB-231 cells were solubilized and incubated with anti-EGFR antibody (**F**)/anti-PDZK1 antibody (**G**) coupled to affinity gel. Co-IP of PDZK1 with EGFR was evident and it was not detectable when IgG was used. **H** EGFR associated with PDZK1 via its PDZ binding motif. Purified GST, GST-EGFR-CT-wild type (WT) and GST-EGFR-CT-mutant (MT, L1042/1063 were mutated to **F**) fusion proteins were used in pull-down assay with lysates of HEK293 cells. Precipitates were subjected to WB with anti-PDZK1 antibody (top panel). Ponceau S staining demonstrated equal loading of the fusion proteins. **I** PDZK1 bound with EGFR via PDZ2/3 domains. GST and GST-EGFR-CT-WT fusion protein were used to pull down His-PDZK1 PDZ1–4 respectively. Precipitates were subjected to WB with anti-His antibody (top panel). Coomassie blue staining demonstrated equal loading of GST and GST-EGFR-CT-WT fusion proteins (bottom panel). **J** PDZK1-WT, but not PDZK1-MT_S2,3 interacted with EGFR. The interaction between PDZK1 and EGFR was detected by Co-IP in MDA-MB-231 cells stably overexpressing Myc-EGFR and co-transfected with PDZK1-WT or PDZK1-MT_S2,3. Data are the representative of three independent experiments (**B**–**J**).

The genes correlated with TNBC development have the greatest potential to be therapeutic targets for sensitising patients to EGFR-targeted therapy. Hence, we identified novel genes affecting TNBC development by bioinformatics analysis. The common differentially expressed genes in both the Gene Expression Omnibus (GEO) GSE21653 microarray dataset and The Cancer Genome Atlas (TCGA) BRCA sequencing dataset were selected. The genes were subsequently subjected to GO annotation, and the correlations of their expression levels with stage, patient survival, migration, invasion, relapse and metastasis were analysed. Finally, PDZK1 was found to be differentially expressed in the GSE21653 and TCGA_BRCA datasets; moreover, PDZK1 was found to be associated with cell proliferation, and its expression was gradually decreased as TNBC stage progressed; PDZK1 expression could predict the prognosis of TNBC patients and was correlated with the degree of cell malignancy in clinical TNBC samples (Fig. 1A, Supplementary Fig. 1A–G). This finding suggested that a low level of *PDZK1* may contribute to TNBC development. In addition, these correlations were not detected in the entire BC patient cohort or in the non-TNBC patients of the cohort (Supplementary Fig. 1H–M), suggesting that PDZK1 specifically affects TNBC development. To observe the correlation between PDZK1 expression and EGFR-TKI sensitivity, we selected the non-TNBC cell line MCF-7, the erlotinib-sensitive TNBC cell line SUM149, the erlotinib-moderately sensitive TNBC cell line SK-BR-3, the erlotinib-resistant TNBC cell lines MDA-MB-231 and MDA-MB-468 [27] and detected their PDZK1 levels. PDZK1 was downregulated in erlotinib-resistant TNBC cells compared with erlotinib-sensitive/erlotinib-moderately sensitive TNBC cells (Fig. 1B), indicating that PDZK1 is correlated with the resistance of TNBC cells to erlotinib.

### PDZK1 is a novel EGFR-binding protein

Interestingly, the GST-PDZK1 pull-down results showed that PDZK1 bound to EGFR (Fig. 1C). Since PDZK1 is an adaptor protein that can regulate the activity of bound proteins [23, 28], we speculated that PDZK1 could regulate EGFR activity by interacting with EGFR. We subsequently verified the interaction between PDZK1 and EGFR. The GST-EGFR-CT-wild type (WT), but not the GST, pulled down PDZK1 (Fig. 1D). Exogenous and endogenous coimmunoprecipitation (Co-IP) of PDZK1 and EGFR in MDA-MB-231 cells was also observed (Fig. 1E–G). The absence of binding between PDZK1 and the GST-EGFR-CT-mutant (MT) demonstrated the specificity of the interaction (Fig. 1H, Supplementary Fig. 2A). Furthermore, the GST pull-down assay results showed that the His-PDZK1-PDZ2 and PDZ3 domains, especially the His-PDZK1-PDZ2 domain, were pulled down by GST-EGFR-CT-WT but not by GST (Fig. 1I, Supplementary Fig. 2B). Co-IP confirmed that the interaction of PDZK1 with EGFR was abolished by PDZ2/3 mutation (YGF to AGA, S2,3; Fig. 1J, Supplementary Fig. 2C). These results revealed that the interaction between EGFR and PDZK1 is mediated by the EGFR-CT and PDZ2/3 domains of PDZK1.

### PDZK1 decreases EGFR levels and EGFR-mediated signalling in TNBC cells via interaction with EGFR

**Fig. 2:**
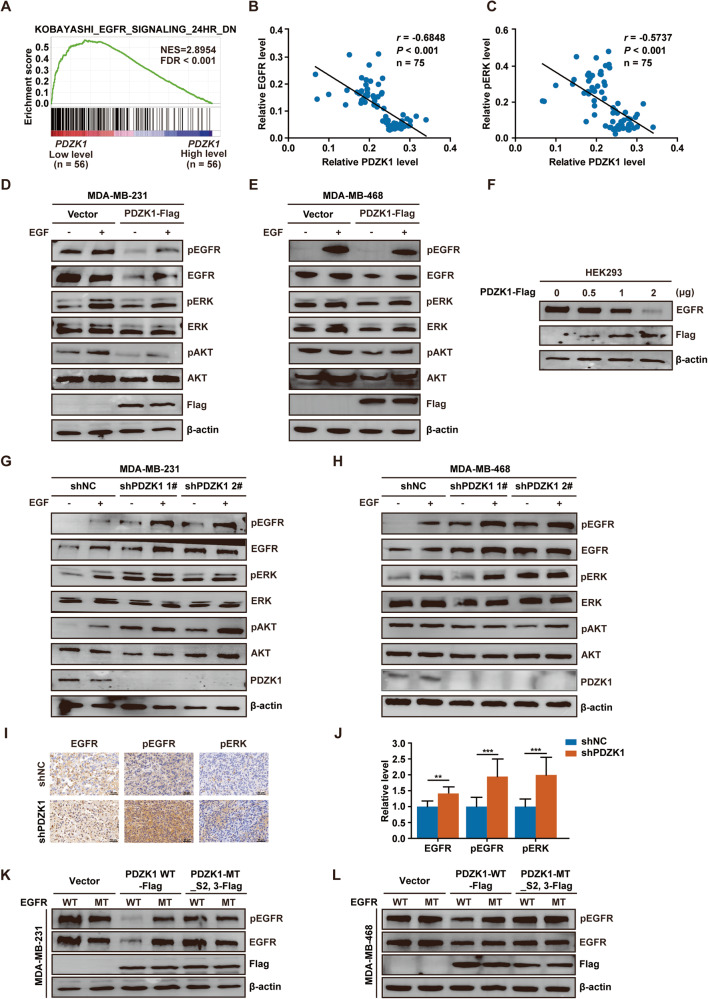
PDZK1 decreases EGFR levels and EGFR-mediated signalling in TNBC via interaction with EGFR. **A** PDZK1 expression was negatively associated with EGFR signalling activation in TNBC. Gene set enrichment analysis (GSEA) profile of the “KOBAYASHI_EGFR_SIGNALING_24HR_DN” gene signature (source: https://www.gsea-msigdb.org/gsea/msigdb/) for *PDZK1* level. According to the median value of the TCGA dataset, the samples were divided into *PDZK1* high/low expression group. NES: Normalised Enrichment Score, FDR: False Discovery Rate. **B**, **C** PDZK1 expression was negatively correlated with EGFR expression and pERK level in TNBC. The expression levels were derived from IHC results of TMA. Data were analysed by Pearson correlation. **D**, **E** PDZK1 overexpression decreased EGFR expression and EGFR-mediated signalling in TNBC cells with/out EGF stimulation. **F** PDZK1 decreased EGFR expression in a dose-dependent manner. **G**, **H** PDZK1 knockdown enhanced EGFR expression and EGFR-mediated signalling in TNBC cells with/out EGF induction. **I**, **J** PDZK1 knockdown enhanced EGFR expression and EGFR-mediated signalling in vivo. Data were presented as mean ± SD with *n* = 8 mice per group and analysed by Student’s two-tailed *t*-test. **P < 0.01 and ***P < 0.001. **K**, **L** PDZK1-WT overexpression decreased the expression and EGFR signalling activation of EGFR-WT. However, PDZK1-WT or PDZK1-MT_S2,3 overexpression could not decrease the expression and EGFR signalling activation of EGFR binding motif mutant. Data are the representative of three independent experiments (**D**–**H**, **K**–**L**).

Since the PDZ protein often regulates the signalling pathway mediated by its binding protein, we speculated that PDZK1 could regulate the EGFR signalling pathway by binding to EGFR. The correlation between EGFR signalling activation and PDZK1 levels was first investigated by gene set enrichment analysis (GSEA). The EGFR pathway activation gene set was highly enriched in the low PDZK1 level group (Fig. 2A), revealing that the PDZK1 level was negatively correlated with EGFR signalling activation in TNBC samples. The correlation between low PDZK1 levels and EGFR pathway activation was further verified by immunohistochemistry (IHC) analysis of tissue microarray (TMA) data (Fig. 2B, C).

EGFR signalling activation is critically dependent on the EGFR level and phosphorylation. Therefore, the impact of PDZK1 on these two variables was determined. PDZK1 overexpression decreased EGFR levels and EGFR-mediated signalling in TNBC cells (Fig. 2D, E). Furthermore, EGFR expression was downregulated with increasing doses of the PDZK1 plasmid (Fig. 2F). These findings suggested that PDZK1 might regulate EGFR levels in a dose-dependent manner. Conversely, EGFR levels and EGFR-mediated signalling were enhanced in TNBC cells with PDZK1 knockdown (Fig. 2G, H). PDZK1 knockdown in vivo relieved the inhibition of EGFR signalling (Fig. 2I, J). Hence, PDZK1 not only downregulated EGFR expression but also inhibited EGFR-mediated signalling in TNBC cells. Moreover, PDZK1-WT overexpression decreased EGFR levels and inhibited EGFR phosphorylation only in cells expressing EGFR-WT but not in those expressing EGFR-MT (Fig. 2K, L). PDZK1-MT_S2,3 overexpression did not affect EGFR levels or signalling (Fig. 2K, L). These results suggested that PDZK1 regulates EGFR-mediated signalling via interaction with EGFR.

### PDZK1 promotes EGFR degradation by enhancing c-Cbl-mediated ubiquitination and inhibits EGFR phosphorylation by hindering EGFR dimerisation in TNBC cells

**Fig. 3:**
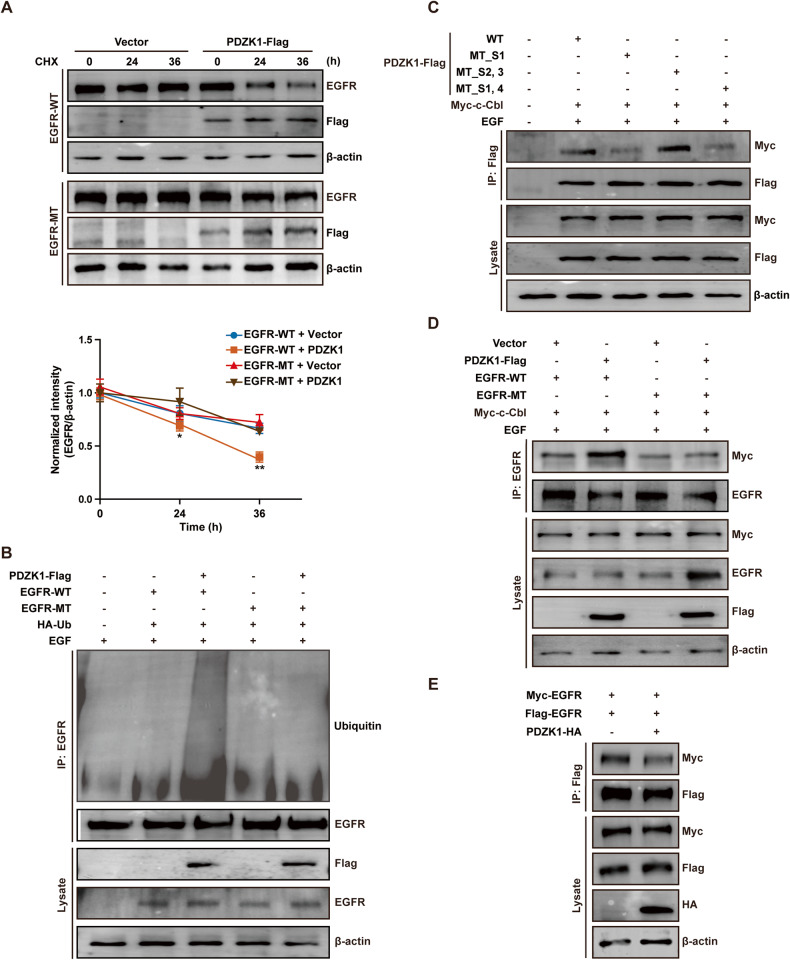
PDZK1 promotes EGFR degradation by enhancing c-Cbl-mediated ubiquitination and inhibits EGFR phosphorylation by hindering EGFR dimerisation in TNBC. **A** PDZK1 overexpression promoted EGFR degradation. Vector and PDZK1 expression plasmid were transfected into MDA-MB-231 cells stably overexpressing EGFR-WT or EGFR-MT for 24 h, then cells were treated with CHX at 0 h, 24 h or 36 h, EGFR protein level was detected by WB. Data were presented as mean ± SD of three independent experiments and analysed by repeated measures ANOVA. *P < 0.05, **P < 0.01. **B** *P*DZK1 overexpression promoted EGF-induced EGFR ubiquitination. MDA-MB-231 cells co-transfected with PDZK1 and EGFR-WT/EGFR-MT were stimulated with EGF, anti-EGFR antibody coupled with beads was used to precipitate lysates and ubiquitinated EGFR was detected by WB. **C** PDZK1 interacted with c-Cbl. PDZK1-WT, PDZK1-MT_S1, PDZK1-MT_S1,4 or PDZK1-MT_S2,3 were transfected into MDA-MB-231 cells stably overexpressing c-Cbl, and the interaction between PDZK1 and c-Cbl was detected by Co-IP. **D** PDZK1 promoted the binding of c-Cbl with EGFR. Myc-c-Cbl plasmid and PDZK1-Flag plasmid were transfected into MDA-MB-231 cells stably overexpressing EGFR-WT or EGFR-MT and were stimulated with EGF. EGFR was precipitated and E3 ubiquitin ligase c-Cbl was detected by WB. **E** Vector or PDZK1-HA expression plasmid were transfected into MDA-MB-231 cells overexpressing Myc and Flag tagged EGFR, and the level of EGFR dimerisation was detected by Co-IP. Data are the representative of three independent experiments (**A**–**E**).

The expression level of a protein can be decreased by inhibiting gene transcription or accelerating protein degradation. However, PDZK1 is not a transcription factor, and the mRNA levels of *PDZK1* and *EGFR* in clinical specimens were not correlated (Supplementary Fig. 3A). *EGFR* mRNA levels were also not decreased by PDZK1 overexpression in TNBC cells (Supplementary Fig. 3B). This finding excluded the possibility that PDZK1 regulates EGFR expression by repressing its transcription. To determine whether PDZK1 regulates EGFR expression by degrading the EGFR protein, we used the protein synthesis inhibitor cycloheximide (CHX). The results showed that exposure of MDA-MB-231 cells to CHX resulted in a faster decrease in expression of the EGFR-WT protein, but not the EGFR-MT protein in PDZK1-overexpressing cells than in vector-transfected cells (Fig. 3A), indicating that PDZK1 promotes EGFR degradation by interacting with EGFR. PDZK1 regulates the ubiquitination of its binding proteins [29, 30]. PDZK1 overexpression enhanced the ubiquitination of EGFR-WT but not EGFR-MT (Fig. 3B). These findings suggested that PDZK1 regulates EGFR degradation by modulating EGFR ubiquitination. As an adaptor, PDZK1 itself is not an E3 ubiquitin ligase. c-Cbl is an E3 ubiquitin ligase of EGFR [31]. Thus, we investigated whether PDZK1 promoted EGFR ubiquitination by enhancing the interaction of EGFR with c-Cbl. To verify this hypothesis, we first demonstrated the interaction of PDZK1 with c-Cbl in MDA-MB-231 cells by Co-IP. Subsequently, the interaction of PDZK1-PDZ1-MT_S1 with c-Cbl was significantly attenuated. PDZK1-PDZ1/4-MT_S1,4, but not PDZK1-PDZ2/3-MT_S2,3 led to a similar attenuation effect (Fig. 3C). These results suggested that the PDZ1 domain of PDZK1 is critical for its interaction with c-Cbl. Co-IP further demonstrated that the binding of c-Cbl to EGFR-WT was promoted by PDZK1 (Fig. 3D). Thus, PDZK1 was identified as a novel ubiquitin binding adaptor of c-Cbl that promotes EGFR degradation by enhancing c-Cbl-mediated EGFR ubiquitination.

In addition, EGFR is a receptor tyrosine kinase. Ligand binding induces dimerisation of the receptor monomer; therefore, intracellular kinase domains (aa 685–953) in two EGFR monomers become closer and *trans*-autophosphorylate one another at the carboxyl terminal (CT) domain (aa 953–1186) in the dimeric state [32, 33]. This process activates the receptor and its downstream molecules. Importantly, the PDZK1 binding DSFL motif of EGFR is located at amino acids 1039–1042 and 1060–1063, which are close to its kinase domain, suggesting that PDZK1 regulates EGFR activity by affecting its dimerisation. Indeed, the results showed that EGFR dimerisation was decreased in PDZK1-overexpressing cells (Fig. 3E), suggesting that PDZK1 inhibits EGFR phosphorylation in TNBC cells by hindering EGFR dimerisation.

### The PDZK1 protein is specifically decreased in TNBC tissues and negatively correlated with the degree of TNBC malignancy

**Fig. 4:**
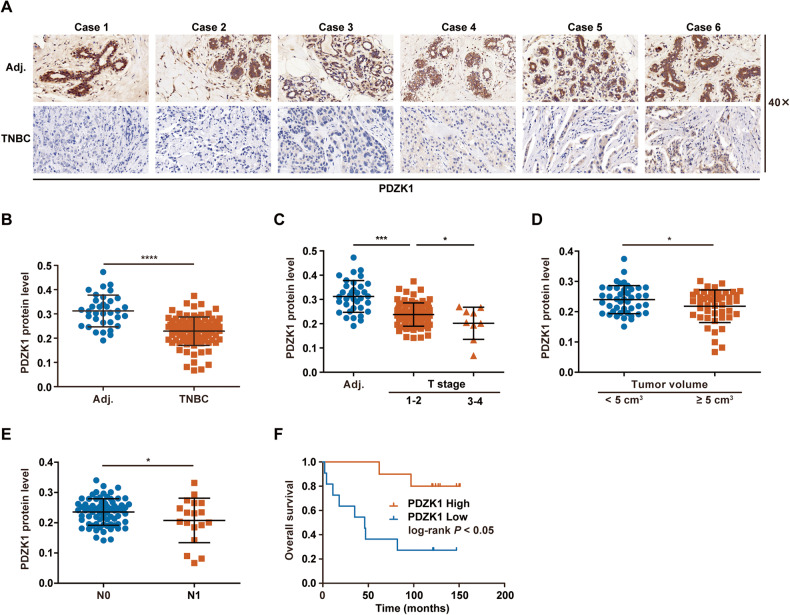
PDZK1 protein is specifically decreased in TNBC tissues and negatively correlated with the degree of TNBC malignancy. **A** PDZK1 protein level was downregulated in TNBC tissues. Representative IHC figure of PDZK1 expression in adjacent normal tissues and TNBC tissues. **B** Scatter plot displaying the expression of PDZK1 in adjacent normal tissues and TNBC tissues. **C** PDZK1 protein level was downregulated gradually as T stage progressed. **D** PDZK1 protein level was correlated with TNBC tumour volume. **E** PDZK1 protein level was correlated with TNBC lymph node metastasis. **F** K-M curve of TNBC patients. TNBC patients were divided into high and low groups according to the median value of PDZK1 protein level. Data were presented as mean ± SD. Data were analysed by Student’s two-tailed *t*-test (**B**, **D**, **E**) and ANOVA (**C**). Survival curves were calculated by Kaplan–Meier methods. *P < 0.05, ***P < 0.001, ****P < 0.0001.

The PDZK1 protein level was found to be decreased in TNBC tissues *vs*. adjacent normal tissues (Fig. 4A, B), and gradually decreased as T stage progressed (Fig. 4C). Moreover, the PDZK1 protein level was negatively correlated with tumour volume (Fig. 4D). These results suggested that abnormal downregulation of the PDZK1 protein might be closely related to TNBC occurrence. TNBC patients with lower PDZK1 levels had a higher rate of lymph node metastasis and shorter overall survival (OS) time (Fig. 4E, F), suggesting that PDZK1 can serve as a predictor of metastasis and a poor prognosis in TNBC patients. Consistent with the results for mRNA levels, this phenomenon occurred only in TNBC tissues. The PDZK1 protein level in non-TNBC tissues was comparable to that in paracancerous tissues and did not change with the progression of TNBC malignancy (Supplementary Fig. 4A–F), suggesting that PDZK1 specifically suppresses TNBC development.

The mechanism underlying the specific downregulation of PDZK1 in TNBC tissues was subsequently explored. A search of the cBioPortal database did not reveal a deletion or mutation in the *PDZK1* gene (Supplementary Fig. 5A). Therefore, the methylation level of the *PDZK1* promoter was examined. The methylation level of the *PDZK1* promoter was not increased in non-TNBC patients, consistent with the absence of changes in its expression level. In contrast, the methylation level of the *PDZK1* promoter was increased in TNBC, possibly explaining the decrease in the PDZK1 level in TNBC (Supplementary Fig. 5B). On this basis, MDA-MB-231 cells were treated with decitabine, an epigenetic drug that inhibits DNA methylation. Decitabine promoted dose-dependent upregulation of PDZK1 (Supplementary Fig. 5C), suggesting that the decrease in PDZK1 protein levels resulted from methylation of the *PDZK1* promoter.

### PDZK1 suppresses TNBC development

**Fig. 5:**
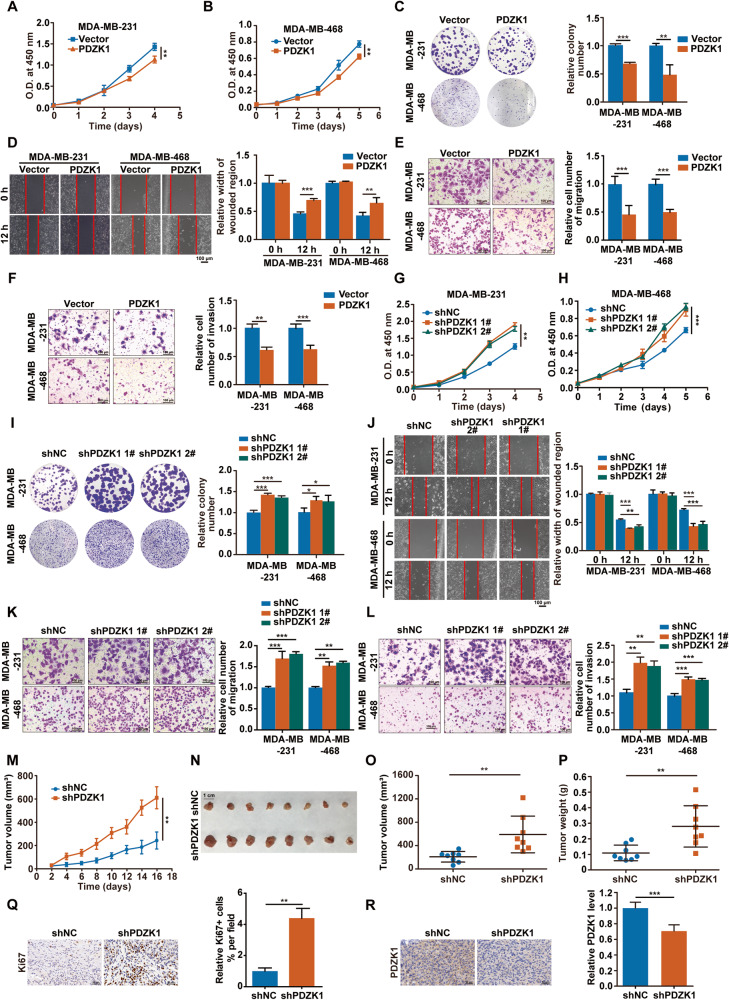
PDZK1 suppresses TNBC development in vitro and in vivo. **A**, **B** PDZK1 overexpression suppressed the viabilities of TNBC cells. Cell viability was detected using CCK8 assay. **C** PDZK1 overexpression suppressed colony formation abilities of TNBC cells. Colony formation ability was detected by plate clone formation assay. PDZK1 overexpression suppressed the migration and invasion abilities of TNBC cells. Wound healing assay (**D**) and Boyden chamber assay were used to detect cell migration (**E**) and invasion (**F**), respectively. **G**, **H** PDZK1 knockdown promoted the viabilities of TNBC cells. shNC, nontarget control. **I** PDZK1 knockdown promoted colony formation abilities of TNBC cells. PDZK1 knockdown promoted the migration and invasion abilities of TNBC cells. Wound healing assay (**J**) and Boyden chamber assay were used to detect cell migration (**K**) and invasion (**L**), respectively. **M** Growth curve of xenograft tumours. **N** Photos of xenograft tumours. **O** The volume of xenograft tumours. **P** The weight of xenograft tumours. IHC results of Ki67 level (**Q**) and PDZK1 protein level (**R**) in xenograft tumour tissues. Data were presented as mean ± SD of three independent experiments. Data were analysed by repeated measures ANOVA (**A**, **B**, **G**, **H**, **M**), Student’s two-tailed *t*-test (**C**–**F**, **O**–**R**) and ANOVA (**I**–**L**). *n* = 8 mice per group (**M**–**R**). **P < 0.01. ***P < 0.001.

The above data raised the possibility that decreased PDZK1 levels in TNBC correlate with TNBC malignancy. However, the influence of PDZK1 on TNBC development has not been determined. To address this issue, PDZK1 was overexpressed or knocked down, and the effect of this intervention on TNBC cell malignancy was observed. In comparison with that in vector-control cells, PDZK1 overexpression inhibited TNBC cell malignancy in vitro (Fig. 5A–F). Conversely, in comparison with negative control cells, cells with PDZK1 knockdown exhibited markedly enhanced TNBC cell malignancy in vitro (Fig. 5G–L). Moreover, PDZK1 knockdown also promoted the proliferation of MDA-MB-231 cells in vivo (Fig. 5M–R). Taken together, these results suggested that PDZK1 is a novel tumour suppressor specific for TNBC.

### Changes in TNBC malignancy induced by PDZK1 overexpression or inhibition are reversed by restoration of EGFR expression or pretreatment with an EGFR kinase inhibitor

**Fig. 6:**
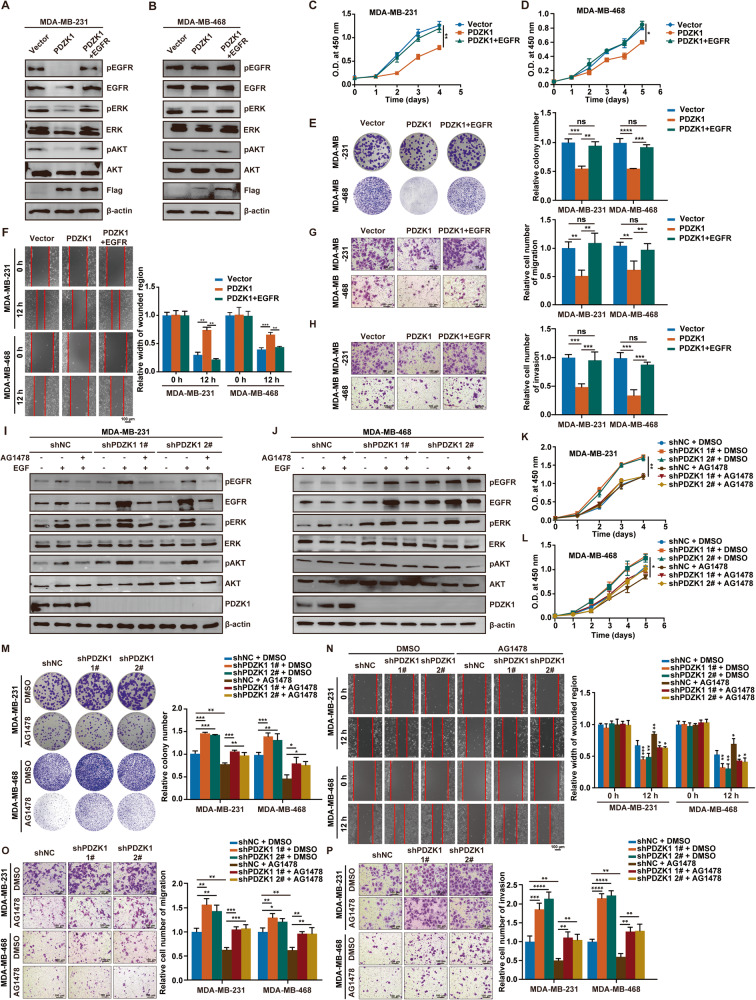
Changes in TNBC malignancy induced by PDZK1 overexpression or inhibition are reversed by restoration of EGFR expression or pretreatment with an EGFR kinase inhibitor. Restoration of EGFR expression reversed the PDZK1-mediated inhibition of EGFR signalling (**A**, **B**) and malignancy (**C**, **H**) of TNBC. Inhibition of EGFR phosphorylation by AG1478 pretreatment reversed the promotion of PDZK1 knockdown on EGFR signalling (**I**–**J**) and malignancy (**K**–**P**) of TNBC. WB data are the representative of three independent experiments (**A**, **B**, **I**–**J**). Other data are shown as mean ± SD of three independent experiments. Data were analysed by repeated measures ANOVA (**C**, **D**, **K**, **L**) and ANOVA (**E**–**H**, **M**–**P**). **P < 0.01, ***P < 0.001. ns, not significant.

To determine whether PDZK1 suppresses TNBC development by regulating EGFR levels and phosphorylation, EGFR levels were restored or EGFR phosphorylation was inhibited in TNBC cells with PDZK1 overexpression or knockdown. Restoring EGFR levels in cells overexpressing PDZK1 reversed the PDZK1-induced inhibition of EGFR signalling and cell malignancy (Fig. 6A–H). Inhibiting EGFR-mediated signalling with the kinase inhibitor AG1478 in PDZK1-knockdown cells reversed the increase in EGFR-mediated signalling and cell malignancy degree induced by PDZK1 knockdown (Fig. 6I–P). This demonstrated that the function of PDZK1 in TNBC cells depended on its inhibition of EGFR expression and phosphorylation.

### PDZK1 sensitizes TNBC cells to erlotinib

The above results showed that PDZK1 is a potential therapeutic target for TNBC and might sensitise TNBC cells to EGFR-targeted therapy. More importantly, in the prescreened TNBC patients with high EGFR expression, the *PDZK1* level gradually decreased as the TNBC stage progressed (Supplementary Fig. 6A). Subsequently, when these patients were divided into two groups based on the median *PDZK1* level, the EGFR signalling pathway was abnormally activated in patients with low *PDZK1* levels, and these patients had poorer prognoses (Supplementary Fig. 6B–D). These findings suggested that PDZK1 might play a suppressive role in the progression of TNBC with high EGFR levels. Therefore, the differential responses of patients with high EGFR expression to EGFR-TKIs may be due to the differential expression of PDZK1, and increasing PDZK1 levels in patients with low PDZK1 expression may sensitise TNBC cells to erlotinib. Taken together, these results suggested that PDZK1 overexpression might sensitise TNBC cells to erlotinib.

**Fig. 7:**
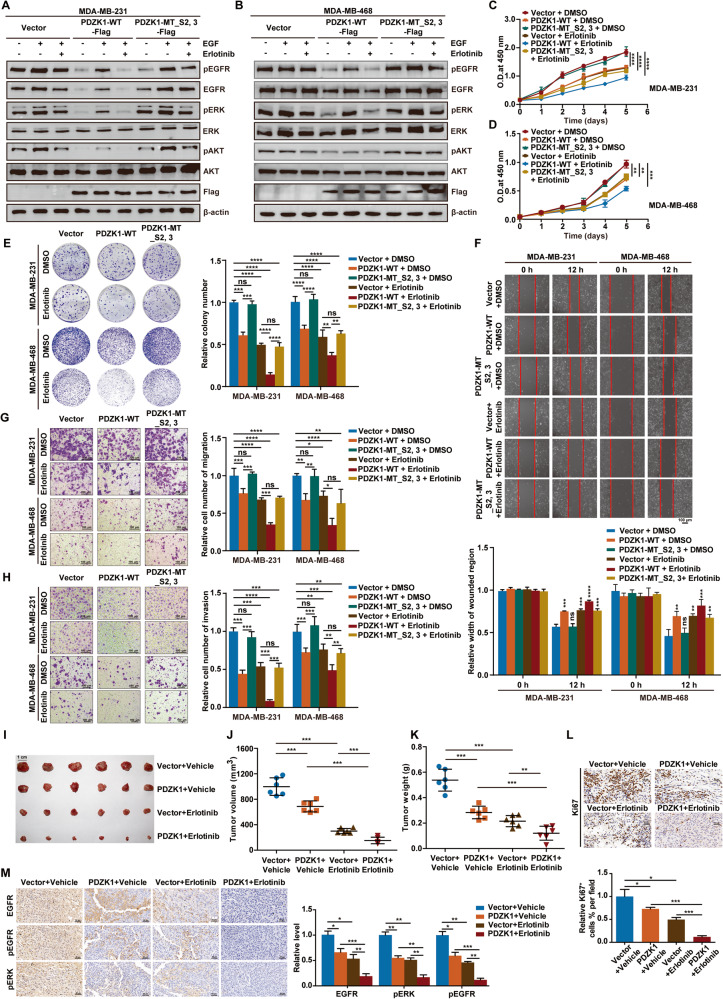
PDZK1 sensitizes TNBC cells to erlotinib in vitro and in vivo. **A**, **B** PDZK1-WT, but not PDZK1-MT_S2,3 overexpression sensitised TNBC cells to erlotinib in inhibiting EGFR-mediated signalling pathway. WB analysis of phosphorylation levels of EGFR and its downstream molecules in TNBC cells stably expressing empty vector or PDZK1-WT or PDZK1-MT_S2,3 combined with erlotinib treatment. All WBs are representative of one of three independent experiments (**A**, **B**). PDZK1 overexpression sensitised TNBC cells to erlotinib in inhibiting cell proliferation (**C**, **D**), colony formation (**E**), migration (**F**, **G**) and invasion (**H**) abilities. The photo (**I**), the volume (**J**) and the weight (**K**) of xenograft tumours, treated with PDZK1 overexpression alone, erlotinib alone or a combination of PDZK1 overexpression with erlotinib (*n* = 6 mice per group). IHC results of Ki67 level (**L**) and EGFR/pEGFR/pERK level (**M**) in xenograft tumour tissues. Data are shown as mean ± SD of three independent experiments. Data were analysed by repeated measures ANOVA (**C**, **D**) and ANOVA (**E**–**H**, **J**–**M**). *P < 0.05, **P < 0.01, ***P < 0.001 and ****P < 0.0001. ns, not significant.

In erlotinib-resistant MDA-MB-231 and MDA-MB-468 cells expressing lower levels of PDZK1, we overexpressed PDZK1-WT or PDZK1-MT by transfecting PDZK1-WT or PDZK1-MT_S2,3 plasmid into cells. Overexpression of PDZK1-WT, but not PDZK1-MT, combined with erlotinib treatment had a stronger inhibitory effect on EGFR signalling and cell malignancy than erlotinib alone (Fig. 7A–H). These findings suggested that PDZK1 overexpression might sensitise TNBC cells to erlotinib in vitro through interaction with EGFR.

**Fig. 8:**
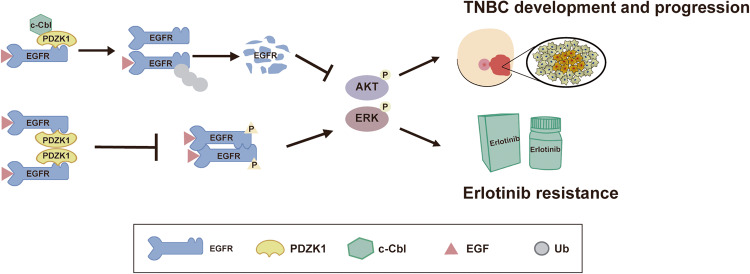
A proposed model for PDZK1 regulating c-Cbl mediated EGFR degradation, EGFR dimerisation and phosphorylation, thereby affecting the progression of TNBC and sensitising TNBC cells to erlotinib.

To investigate whether PDZK1 overexpression could sensitise TNBC cells to erlotinib in vivo, we further observed the effects of erlotinib treatment on tumour xenografts overexpressing PDZK1. The results showed that erlotinib more effectively suppressed tumour proliferation in vivo when PDZK1 was overexpressed (Fig. 7I–M). Hence, we propose that PDZK1 degrades EGFR and blocks EGFR phosphorylation to affect TNBC development and erlotinib sensitivity (Fig. 8).

## Discussion

This study demonstrated for the first time that the downregulation of PDZK1 in TNBC cells relieved its inhibition of the EGFR pathway and induced resistance to EGFR-targeted therapy. PDZK1 overexpression sensitised TNBC cells to erlotinib by inhibiting EGFR signalling. These results highlighted the potential of PDZK1 as an important therapeutic target for TNBC patients highly expressing EGFR. The results may be helpful for the precise treatment of TNBC patients, and EGFR-targeted therapy may be effective in TNBC patients with high levels of PDZK1. A combination of a low dose of an EGFR-targeted drug and PDZK1 overexpression might be used to treat TNBC.

PDZK1 was identified as a PDZ adaptor protein that forms an apical/subapical membrane scaffold that binds sodium-hydrogen exchanger 3 (NHE3) and facilitates the downregulation of its activity in response to cAMP and the activation of protein kinase A [34]. PDZK1 participates in tumour development by interacting with multiple kinds of proteins, including multidrug resistance-associated protein 2 (MRP2), and regulating their activities [35]. In different microenvironments, PDZK1 may bind to different partners to exert diverse effects. Recently, PDZK1 was reported to play a role in renal cancer and gastric cancer progression by regulating SHP-1 and PTEN phosphorylation, respectively [23, 28, 36]. For breast cancer, *PDZK1* is a new susceptibility locus [37]. In non-TNBC cells, PDZK1 exerts a tumour-promoting effect by mediating the formation of the oestrogen receptor α (ERα)/EGFR/Src complex [30, 38]. However, its role and mechanism in TNBC have not been elucidated.

Our screening results showed that PDZK1 might participate in the development and progression of TNBC. Moreover, TNBC cells are negative for ERα, and the mechanism of EGFR/ERα/Src complex formation in non-TNBC cells does not occur in TNBC cells. This triggered our interest. We found a novel suppressive role of PDZK1 in TNBC. Mechanistically, PDZK1 interacts with EGFR to regulate EGFR signalling simultaneously by degrading EGFR and blocking EGFR-mediated signalling. The PDZ protein Erbin can regulate EGFR ubiquitination and protein stability by interacting with c-Cbl through its PDZ domain [39]. The PDZ protein MAGI3 acts as a ubiquitin binding adaptor to promote substrate protein degradation [40]. This study further showed that the PDZ protein PDZK1 is another ubiquitin binding adaptor and promotes the binding of c-Cbl to EGFR, leading to EGFR protein degradation. This is a novel mechanism of EGFR degradation, and the PDZ1 domain of PDZK1 is the key domain for its interaction with c-Cbl. With respect to blocking EGFR-mediated signalling, PDZK1 inhibited EGFR signalling via a new mechanism different from that of EGFR-TKIs. PDZK1 overexpression and EGFR-TKI treatment synergistically inhibited EGFR signalling. These results expand our understanding about the role of PDZK1 and how EGFR activity is regulated in TNBC.

This work established that PDZK1 plays a tumour-suppressing role in TNBC by inhibiting EGFR signalling. The same protein can play distinct roles in different breast cancer subtypes through different mechanisms [41]. For example, FOXF2 suppressed tumour growth by inhibiting abnormal DNA replication and cell proliferation in non-TNBC cells, and induced carcinogenic effects by upregulating genes that promote epithelial-to-mesenchymal transition (EMT) in TNBC [41]. This finding further supported the reliability of our results regarding the context-dependent role of PDZK1 in TNBC and non-TNBC.

EGFR-targeted drugs are more effective in patients with *EGFR* mutations [42, 43]. The TCGA_BRCA dataset showed that mutations in the *EGFR* gene are rare in TNBC tissues (Supplementary Fig. 7). This may be the basis for the lower response rate of TNBC to EGFR-targeted therapy. This work suggested that TNBC patients with wild-type EGFR may respond more favourably to combination therapy targeting both PDZK1 and EGFR than to EGFR-targeted therapy alone. Enhancing the PDZK1-EGFR interaction may also be a direction for an effective response in TNBC patients. In addition, the PDZ binding motif of EGFR is very important for the regulation of EGFR activity by PDZK1. *EGFR* mutations (Q1067G, R1068G) close to the PDZ binding motif (1037–1065) have been identified in tumour samples [44]. Whether these *EGFR* mutations are present in TNBC and affect the regulation of the EGFR pathway by affecting its binding with PDZK1 deserves further study.

In conclusion, this study provides the first evidence of the tumour-suppressing role of PDZK1 in TNBC. PDZK1 may be a potential therapeutic target for TNBC. Combining erlotinib treatment with PDZK1 overexpression might lead to more effective treatment of TNBC.

## Materials and methods

### Bioinformatics

GEO GSE21653 data and TCGA_BRCA_IlluminaHiSeq_RNASeq V2 data were from https://www.ncbi.nlm.nih.gov/geo/query/acc.cgi?acc=GSE21653 and https://www.synapse.org/#!Synapse:syn1446183, respectively. Clinical data were from https://www.cbioportal.org/study/summary?id=brca_tcga.

DAVID 6.7 online analysis tool (https://david.ncifcrf.gov) was used to map the Gene Ontology (GO). The correlation of PDZK1 level with activated gene set of EGFR pathway (“KOBAYASHI_EGFR_SIGNALING_24HR_DN” gene signature, source: https://www.gsea-msigdb.org/gsea/msigdb/) was analysed by GSEA (http://software.broadinstitute.org/gsea/) as described previously [45].

### Cells

MCF-7, SK-BR-3, MDA-MB-231 and MDA-MB-468 cell lines obtained from Cell Resource Centre, Institute of Basic Medical Sciences, CAMS/PUMC (Beijing, China) and HEK293 cell line from FuHeng BioLogy (Shanghai, China) were cultured in DMEM medium (Gibco, MA, USA). SUM149 cells obtained from National Collection of Authenticated Cell Cultures (Shanghai) was cultured in Ham’s F-12 medium (Gibco) supplemented with 1 μg/mL hydrocortisone and 5 μg/mL insulin (MedChemExpress, NJ, USA). Both media contained 10% FBS (Shanghai Life-iLab Biotech) and 1% penicillin-streptomycin (HyClone, Logan, UT, USA). Cells were cultured at 37 °C in an incubator with 5% CO_2_.

Cell was transfected using Lipofectamine LTX (Invitrogen, Waltham, USA). For stable transfection, cells were screened with 400 µg/mL G418 (MedChemExpress) or 2.5 µg/mL puromycin (Sigma, St. Louis, USA). All cells were authenticated by short tandem repeat DNA fingerprinting and tested for mycoplasma before using.

For signalling pathway detection, cells were serum-starved for 12 h and pretreated with 10 nM erlotinib (MedChemExpress) for 4 h or 0.25 µM EGFR phosphorylation inhibitor AG1478 (MedChemExpress) for 30 min and then cells were treated with 100 ng/mL EGF for 30 min. For malignant phenotype detection, cells were cultured with 2.5 μM AG1478 or 1.5 μM erlotinib. For protein degradation analysis, CHX (MedChemExpress) was added in 50 mM.

### Plasmids and fusion proteins

PDZK1 overexpression and knockdown plasmids were a kind gift from Dr. Beard (University of Adelaide) [46]. pBK-CMV-Flag-EGFR plasmid was kindly provided by Dr. Rockman. pcDNA3.1-PDZK1-Flag PDZ domain mutants (MTs, YGF in PDZ domain was mutated to AGA), pBK-CMV-Flag-EGFR-MT (key residues L1042/1063 of PDZ binding motif were mutated to F) and pcDNA3.1-Myc-c-Cbl plasmid were from Zeqiong Biotechnology Co., Ltd (Changsha, China).

The Glutathione S-transferase (GST)-tagged EGFR-CT (aa 1022–1186) wild type (GST-EGFR-CT-WT), EGFR-CT-MT and full length PDZK1, His-tagged PDZK1 PDZ1–4 plasmids were generated via PCR amplification, then inserted into pGEX-4T-1 and pET-28a, respectively and verified by DNA sequencing. IPTG (Beyotime, Shanghai) was used to induce the expression of fusion proteins.

### Western blotting, GST Pull-down and co-immunoprecipitation assays

WB was performed and analysed as described before [36]. Equal amounts of protein were electrophoresed on 10% SDS-PAGE gels and were then transferred onto PVDF membranes (Millipore, MA, USA), which were soaked for 2 h in 5% skim milk. Subsequently, the membranes were probed with the indicated primary antibodies (the antibodies were listed in Supplementary Table S1). Then, the PVDF membranes were incubated with corresponding secondary antibodies (the antibodies were listed in Supplementary Table S1) for 60 min. After washing three times (15 min each time), the targeted proteins were visualised using enhanced chemiluminescence reagent (Millipore).

GST Pull-down was performed as previously reported [47]. In brief, equal amounts of GST fusion proteins (conjugated on beads) were incubated with 1 mL cell lysates with end-over-end rotation at 4 °C for 3 h. The beads were washed 1 time with washing buffer I [10 mM 2-[4-(2-hydroxyethyl) piperazin-1-yl] ethanesulfonic acid (HEPES), 50 mM NaCl, 5 mM EDTA, 1 mM benzamidine, 0.1% Tween 20, and 3% bovine serum albumin, then washed 4 times with washing buffer II (buffer I without 3% bovine serum albumin). The proteins were eluted from the beads with SDS-PAGE sample buffer, resolved via SDS-PAGE, and detected by western blotting.

Co-IP were performed as previously reported [47]. Briefly, lysates of transfected cells were solubilized, clarified, and then incubated with 10 μL of antibodies (the antibodies were listed in Supplementary Table S1) with protein A/G-agarose for 5 h with end-over-end rotation at 4 °C. After five times washes with 1 mL ice-cold PBS buffer, the immunoprecipitated proteins were eluted from the beads with SDS-PAGE sample buffer.

### Tissue microarrays, xenograft tumour tissues and immunohistochemistry

TMAs (Supplementary Table S2 and Table 1) were obtained from Shanghai Outdo Biotech Co., Ltd. (SOBC) and were used for IHC and OS analyses. TNBC patients were staged according to the TNM staging system of the American Joint Committee on Cancer staging system. Complete clinic-pathological follow-up data of the TNBC patients were collected. None of the patients recruited to this study received any pre-operative treatments. Informed consent was obtained for all patients recruited. Xenograft tumour tissues from nude mice were formalin fixed and paraffin embedded.

**Table 1:** Clinicopathologic characteristics of breast cancer samples.

	TNBC	Non-TNBC
Characteristics	*n* = 96 (%)	*n* = 124 (%)
Age (years) median, [min-max]	55.48 [32–82]	52 [42–83]
T Stage		
1–2	82 (85.4)	113 (91.1)
3–4	9 (9.4)	10 (8.1)
Missing	5 (5.2)	1 (0.8)
Lymph node status		
Negative	72 (75.0)	47 (37.9)
Positive	18 (18.7)	75 (60.5)
Missing	6 (6.3)	2 (1.6)
TNM stage		
I–II	84 (87.5)	81 (65.3)
III–IV	12 (12.5)	41 (33.1)
Missing	0 (0)	2 (1.6)
Histological grade		
1–2	61 (63.5)	102 (82.3)
3–4	35 (36.5)	22 (17.7)
Missing	0 (0)	0 (0)

IHC was performed and analysed as described before [36]. Briefly, the tissue paraffin sections were deparaffinized, and endogenous peroxidase activity was blocked by incubation with 3% H_2_O_2_. The blocked sections were incubated with antibody (the antibodies were listed in Supplementary Table S1) at 4 °C overnight and then incubated with a biotin-labelled secondary antibody. The sections were stained with 3,3′-diaminobenzidine. Finally, the sections were counterstained using hematoxylin and fixed.

### Cell-based assays

Cell-based assays were performed as previously described [36, 48]. Briefly, for cell proliferation assay, cells were seeded in 96-well plates (3000 cells per well). Plates were then incubated for 4–5 days, and viable cells were analysed with Cell Counting Kit-8 (Dojindo, Kumamoto, Japan) by using a Enspire, microplate reader (Perkin Elmer, Waltham, MA, USA) at 450 nm; For colony formation assay, TNBC cells (500 cells/well) was measured in a six-well plate after 12 days. The colonies were fixed and stained with 0.05% crystal violet (Beyotime, China) before counting; For wound healing assay, cells were plated in six-well dishes and the cell monolayers were scraped using a P-20 micropipette tip. The width of wounded region was monitored and measured; For cell migration and invasion assays, 2.5 × 10^4^ TNBC cells in serum-free media were seeded into the upper chamber of Transwell containing 8 µm pore polycarbonate membrane (for migration) or Transwell having a polycarbonate membrane coated with thin basement membrane (Corning® Matrigel® Basement Membrane Matrix, LDEV-free, for invasion). The lower chamber was filled with 10% FBS RPMI. After 24 h of incubation at 37 °C, cells remaining on the top side of the membrane were removed using a cotton swab, and migrating/invasive cells were fixed, stained, photo graphed and counted.

### In vivo xenograft formation assay

Female BALB/c nude mice (~4 weeks old) were from Beijing Vital River Laboratory Animal Technology Co., Ltd. For the study of PDZK1 influencing TNBC phenotype, MDA-MB-231 cells (2.5 × 10^5^) were injected into the bilateral axilla of nude mice which were randomly divided into two groups (*n* = 8 for each group). The volume of the xenografts was assessed every two days. After 4 weeks, the tumours were harvested. For the study of PDZK1 in sensitising TNBC cells to erlotinib, MDA-MB-231 cells (6 × 10^5^) were subcutaneously injected into the flank of nude mice. When the tumour volume reached >75 mm^3^, mice were randomised into four groups (*n* = 6 for each group). Erlotinib (40 mg/kg) or vehicle was given every two days by intratumoral injections for approximately 4 weeks. Tumours were monitored every two days. After 3 days of the last injection, the mice were sacrificed and the volume/weight of their tumours were measured. Power analysis and sample size (NCSS-PASS, https://www.ncss.com/) software were used to calculate the required sample size for this study. For animal studies, no blinding was done.

### Statistics

Statistical analyses were performed using Graphpad Prism 8 and IBM SPSS 23. Pearson correlation analysis was used. The *t*-test were used to analyse the significance between two groups. Comparisons among multiple groups were performed with one-way ANOVA followed by Bonferroni post-hoc tests. The proliferation curve results were analysed with repeated measures ANOVA. The data were presented as mean ± SD. The log-rank test for the generated KM curve was conducted. Differences were considered significant when *P* < 0.05.

### Supplementary information

Additional file 1 Supplementary Tables

Additional file 2 Supplementary data-1

Checklist

Original Data File

## Data Availability

The datasets presented in this study can be found in online repositories. The names of the repository/repositories and accession number(s) can be found in the article.
