# Supplementary material for: PDZK1 suppresses TNBC development and sensitizes TNBC cells to erlotinib via the EGFR pathway
Source: Cell Death Dis. 2024 Apr 12;15(3):199. doi: 10.1038/s41419-024-06502-2 (PMC11009252; doi:10.1038/s41419-024-06502-2)
Supplement: Supplementary file 2 — Additional file 2 Supplementary data-1 [file 41419_2024_6502_MOESM2_ESM.doc]

**SUPPLEMENTARY MATERIALS AND METHODS**

**Cell treatment**

For methylation analysis, 5-Aza-2′-deoxycytidine (5-AZA-CdR, decitabine, S1200, Selleck) was added to the culture medium at different concentration for 24 h.

**Reverse transcription and quantitative real-time PCR (qRT-PCR)**

Total RNA was isolated with TRIzol reagent (TianGen, Beijing, China). cDNA was synthesized from 2 μg RNA using a Quant script RT Kit (Promega, WI, USA), according to the manufacturer's instructions. Quantitation of all gene transcripts was performed by qRT-PCR using Power SYBR Green PCR Master Mix and an ABI PRISM 7500 sequence detection system (Applied Biosystems, Foster City, CA). The following specific primers were used: GADPH forward, 5′-GAAGGTGAAGGTCGG AGTC-3′ and reverse, 5′-GAAGATGGTGATGGGATTTC-3′; EGFR forward, 5′- TGGTCAAGTGCTGGATGATAGA-3′ and reverse, 5′- ACGGTAGAAGTTGGAGT CTGTA-3′. Relative expression was calculated according to the ΔCt method with normalization to GADPH.

**Data availability**

The public data analyzed in this study were obtained from TCGA at TCGA_BRCA. GSEA gene sets GOBP_POSITIVE_REGULATION_OF_CELL_ PROLIFERATION_INVOLVED_IN_KIDNEY_DEVELOPMENT, WU_CELL_MIGRATION, ANASTASSIOU_MULTICANCER_INVASIVENESS_ SIGNATURE, YAGI_AML_RELAPSE_PROGNOSIS, ZUCCHI_METASTASIS_ UP, KOBAYASHI_EGFR_SIGNALING_24HR_UP and HALLMARK_PI3K_ AKT_MTOR_SIGNALING were from https://www.gsea-msigdb.org/gsea/msigdb/, and analyzed by GSEA (http://software. broadinstitute.org/gsea/).

**SUPPLEMENTARY FIGURES AND LEGENDS**

**
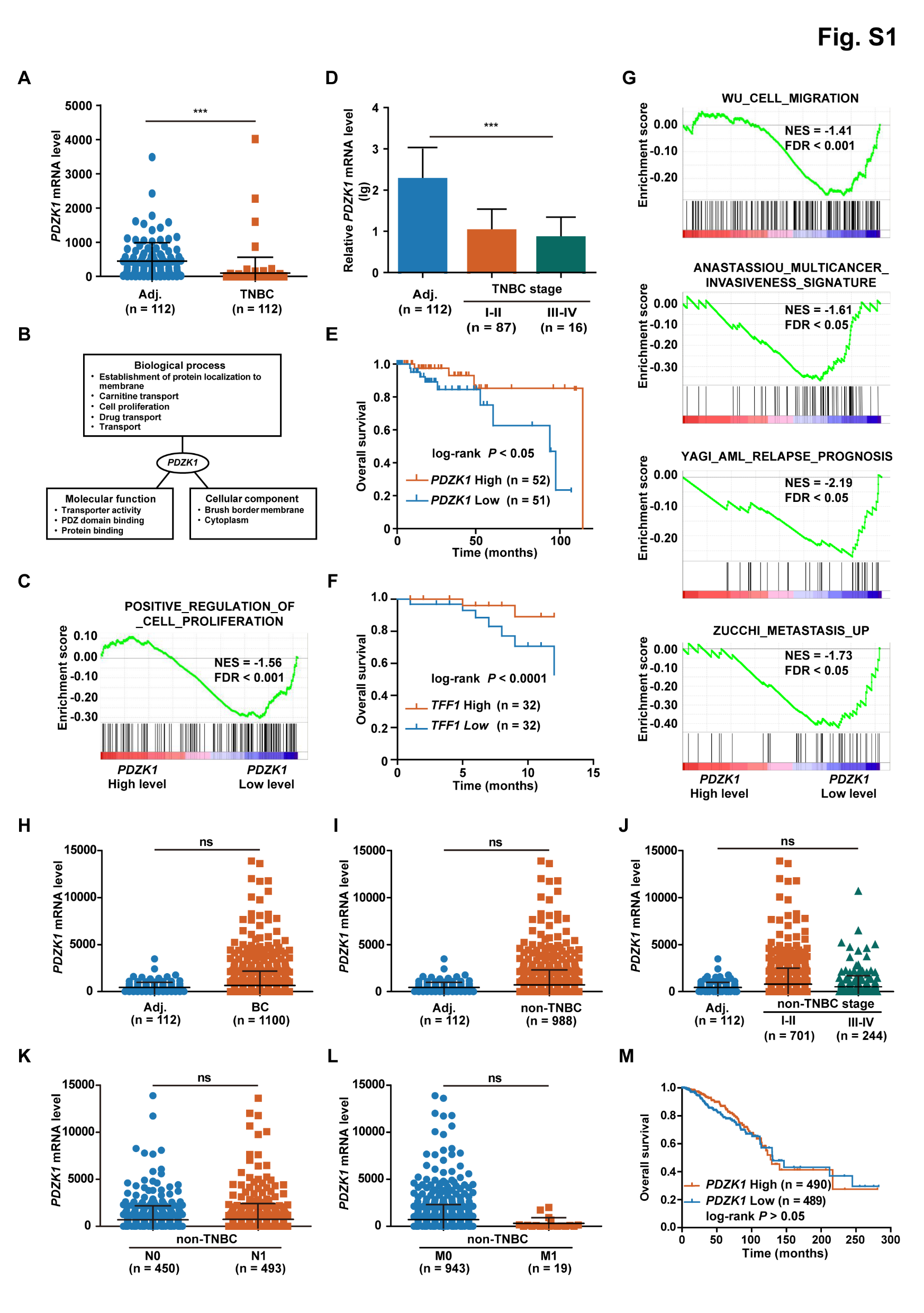
**

**Supplementary Fig. 1 *PDZK1* mRNA level is downregulated in TNBC tissues and negatively correlated with TNBC malignant phenotypes.** **A** *PDZK1* mRNA level was downregulated in TNBC samples. Scatter plot displaying the expression of PDZK1 in adjacent normal tissues and TNBC tissues. *P* value was derived from independent sample two tailed *t*-test. ****P* < 0.001. **B** GO analysis of PDZK1. **C** *PDZK1* downregulation was correlated with cell proliferation in TNBC patients. Enrichment plots of gene expression signatures for cell proliferation according to *PDZK1* mRNA expression levels by gene set enrichment analysis (GSEA) of TCGA_BRCA dataset. Samples were divided into high and low *PDZK1* expression groups according to median value. False discovery rate (FDR) gives the estimated probability that a gene set with a given normalized ES (NES) represents a false-positive finding. **D** *PDZK1* mRNA level was downregulated gradually as stage progressed. *P* value was derived from ANOVA. ****P* < 0.001. **E** Kaplan–Meier (K-M) curve of the TCGA data. Patients were divided into high and low groups according to the median value of *PDZK1* level. **F** Kaplan–Meier (K-M) curve of the TCGA data. Patients were divided into high and low groups according to the median value of *TFF1* level. **G** Enrichment plots of TNBC phenotype gene sets according to *PDZK1* mRNA expression levels by GSEA of TCGA_BRCA dataset. Samples were divided into high and low PDZK1 expression groups according to median value. **H** *PDZK1* mRNA level showed no difference between adjacent normal tissues and all breast cancer tissues. Scatter plot for *PDZK1* mRNA level in normal (*n* = 112) and breast cancer (*n* = 1100) tissues (TCGA_BRCA dataset). Significance between the two populations was determined with independent sample two tailed *t*-test. ns, not significant. **I** *PDZK1* mRNA level showed no difference between adjacent normal tissues and non-TNBC tissues. Scatter plot displaying the expression of PDZK1 in adjacent normal tissues and non-TNBC tissues. *P* value was derived from independent sample two tailed *t*-test. **J** *PDZK1* mRNA level did not change as non-TNBC stage progressed. Scatter plot displaying the expression of PDZK1 in adjacent normal tissues and non-TNBC tissues. *P* value was derived from ANOVA. **K** *PDZK1* mRNA level had no difference between non-TNBC patients with and without lymph node metastasis. **L** *PDZK1* mRNA level had no difference between non-TNBC patients with and without distant metastasis. *P* value was derived from independent sample two tailed *t*-test in A–E. **M** Overall survival of *PDZK1* mRNA level showed no difference between non-TNBC patients. Patients were divided into high and low groups according to the median value of *PDZK1* level.


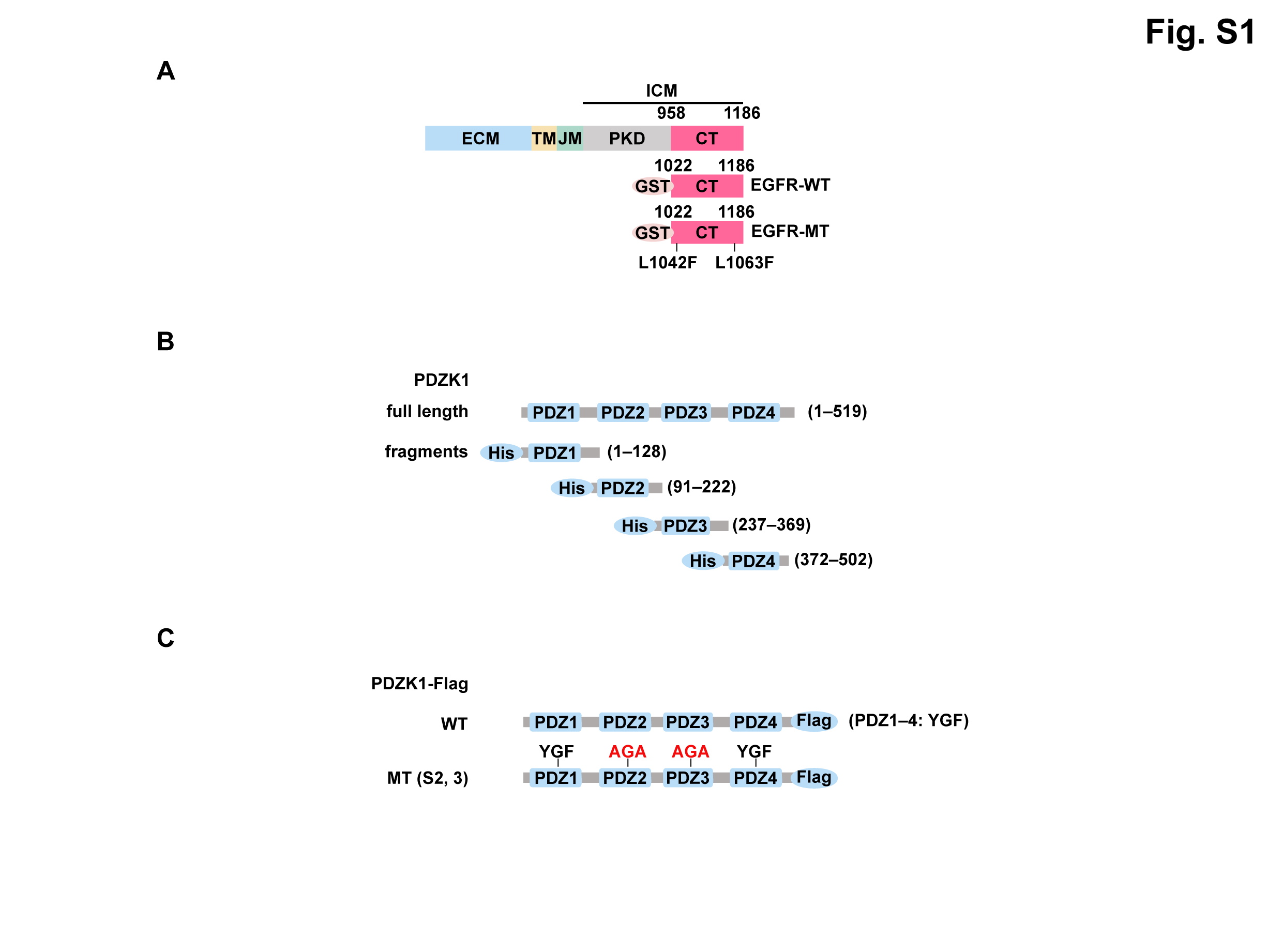


**Supplementary Fig. 2** **Schematic diagram showing design and use of constructs for EGFR and PDZK1. A** Pattern diagram of EGFR full length, GST-EGFR-CT-WT and GST-EGFR-CT-MT. ECM: extracellular module, TM: transmembrane helix, JM: juxtamembrane segment, ICM: intracellular module, PKD: protein kinase domain; CT: C-terminal, autophosphorylation domain. **B** PDZK1 full length and truncated fragment pattern diagram. **C** PDZK1 wild type and PDZK1 mutant pattern diagram.


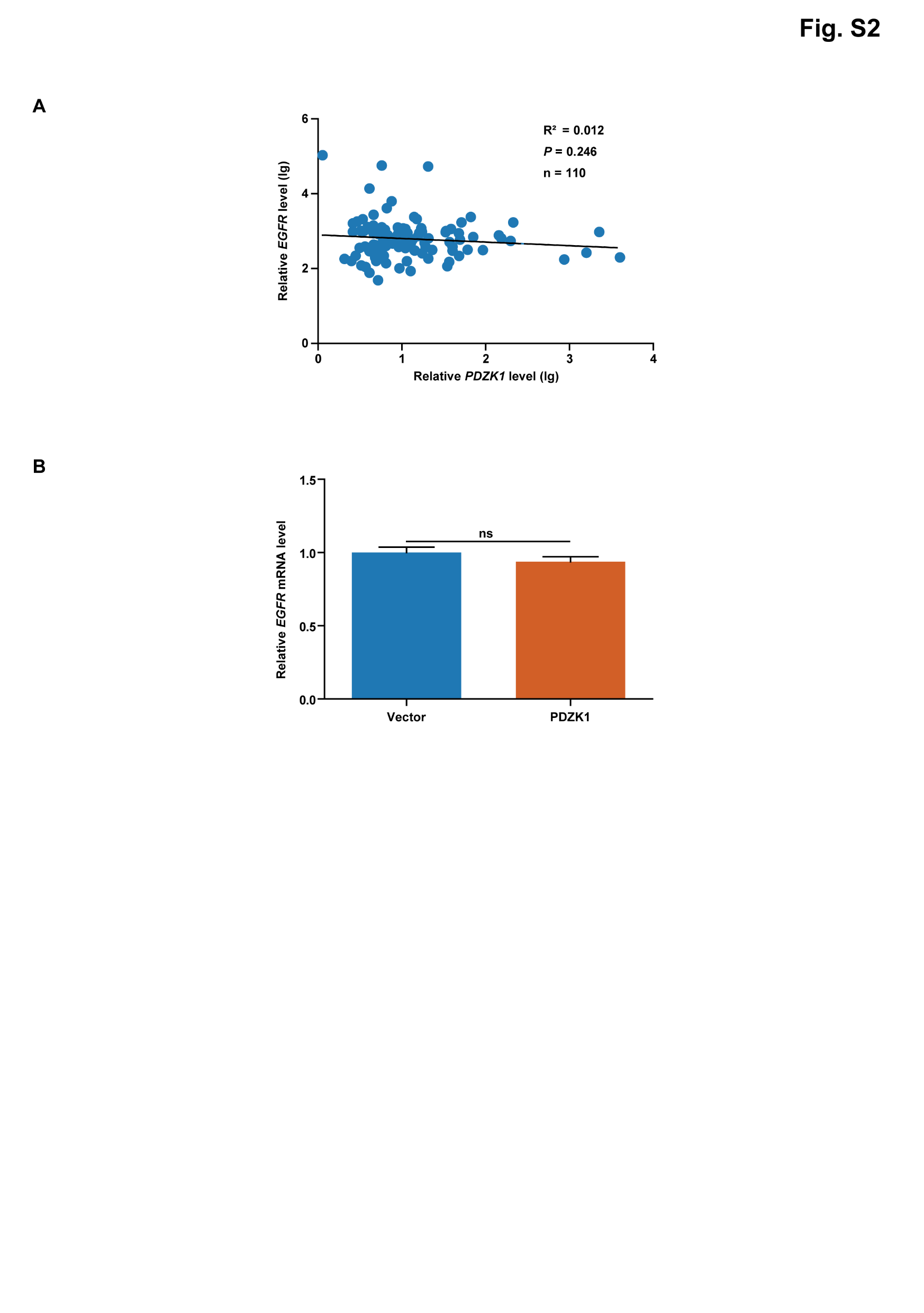


**Supplementary Fig. 3 PDZK1 regulates EGFR expression not by repressing its transcription. A** *PDZK1* mRNA levels in TNBC tissues are not associated with *EGFR* mRNA levels. Pearson correlation and linear regression analysis were used (Pearson Coefficient = -0.112, *P* = 0.246, R^2^ = 0.012). **B** qRT-PCR results showed that PDZK1 overexpression did not induce the decrease of *EGFR* level**.** ns, not significant.

**
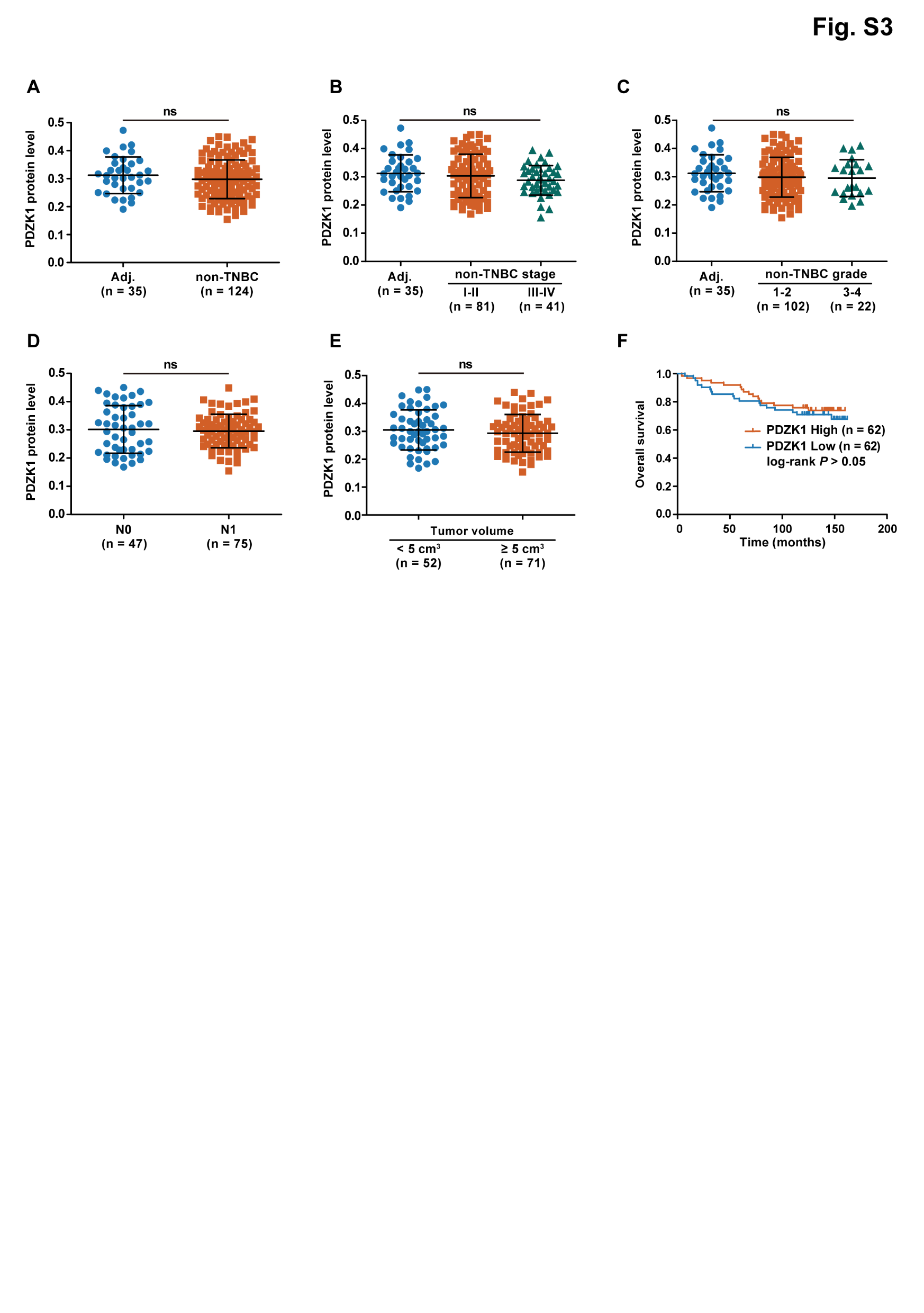
**

**Supplementary Fig. 4 PDZK1 protein level shows no difference between adjacent normal tissues and entire breast cancer tissues/non-TNBC tissues and is not correlated with non-TNBC malignant phenotypes.** **A** PDZK1 protein level had no difference between non-TNBC tissues and adjacent normal tissues. Scatter plot displaying the expression of PDZK1 in non-TNBC tissues and adjacent normal tissues. *P* value was derived from independent sample two tailed *t*-test. ns, not significant. **B** PDZK1 protein level did not change as non-TNBC stage progressed. ns, not significant. **C** PDZK1 protein level did not change as non-TNBC grade progressed. *P* value was derived from ANOVA in B–C. ns, not significant. ns, not significant. **D** PDZK1 protein level had no difference between non-TNBC patients with and without lymph node metastasis. ns, not significant. **E** PDZK1 protein level was not correlated with tumor volume in non-TNBC patients. *P* value was derived from independent sample two tailed *t*-test in D–E. ns, not significant. **F** K-M overall survival curve of the non-TNBC patients. Non-TNBC patients were divided into high and low groups according to the median value of PDZK1 protein level.

**
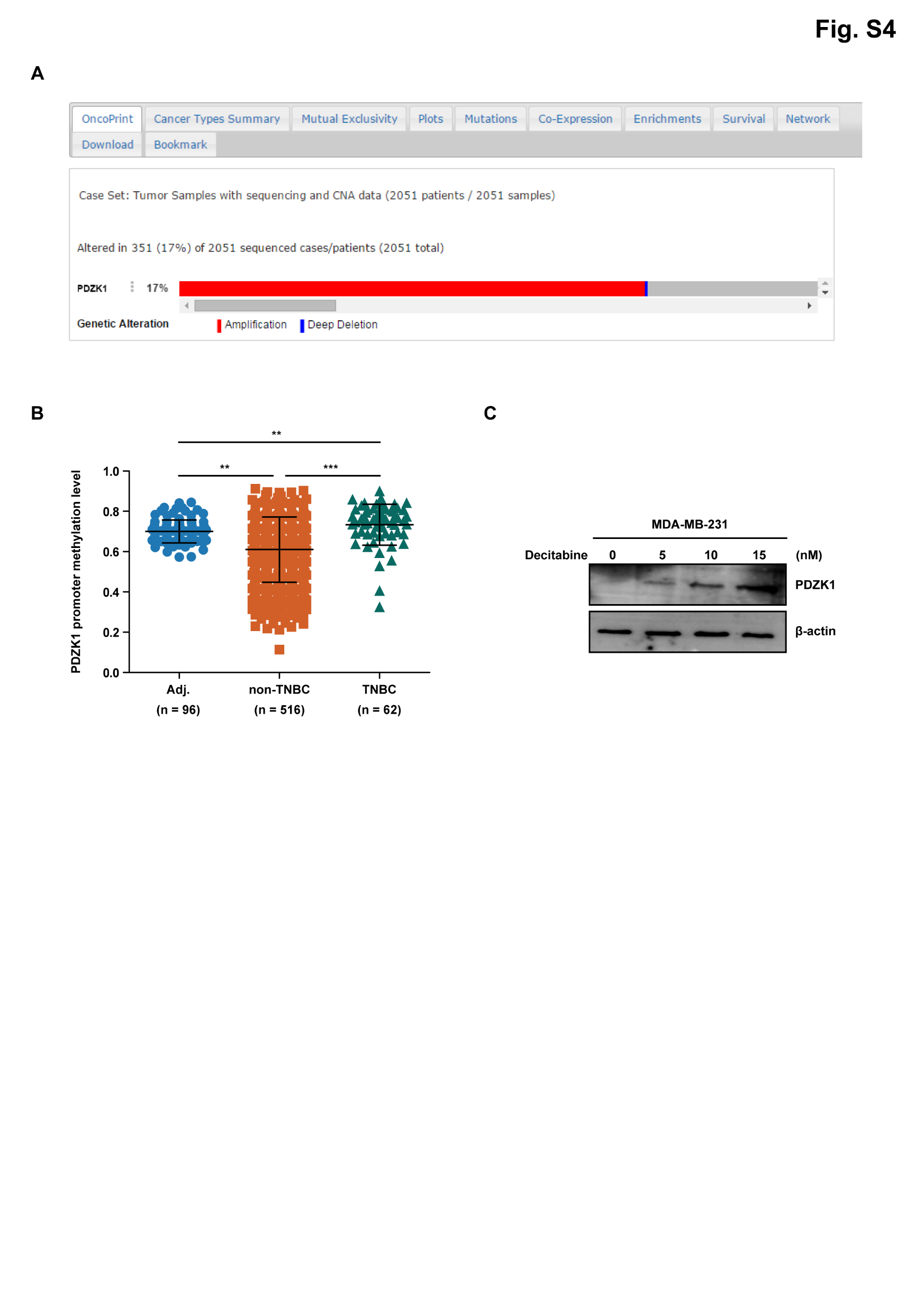
**

**Supplementary Fig. 5 PDZK1 downregulation results from increased methylation of *PDZK1* promoter. A** TCGA cBioportal result revealed that *PDZK1* gene was not amplified or mutated. **B** Scatter plot of the methylation levels of *PDZK1* promoter in adjacent normal breast tissues, non-TNBC and TNBC tissues. *P* value was derived from independent sample two tailed *t*-test. **C** PDZK1 protein level was detected by WB after treating MDA-MB-231 cell with increasing dose of methylation inhibitor decitabine. ***P* < 0.01, ****P* < 0.001.

**
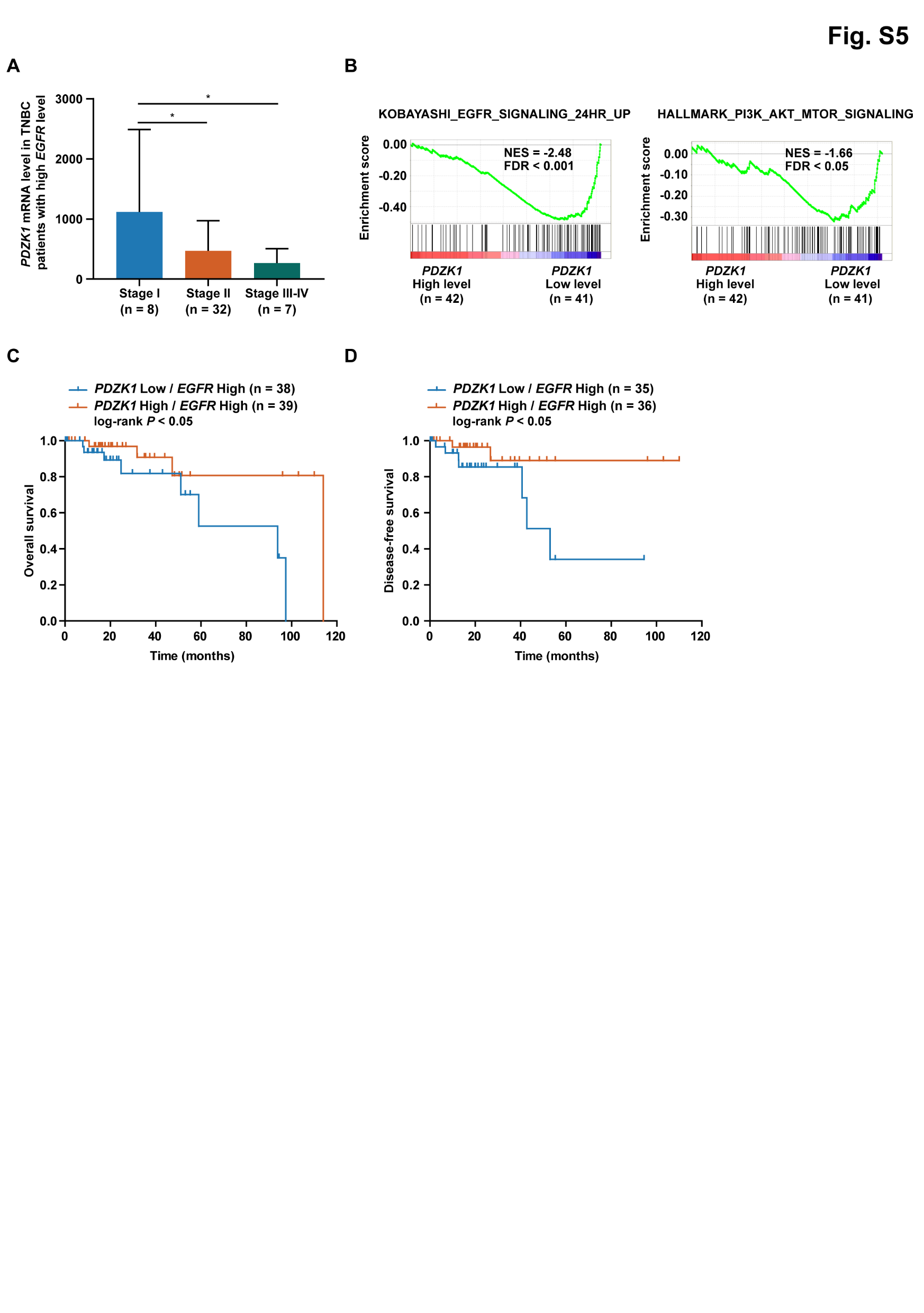
**

**Supplementary Fig. 6 PDZK1 levels are significantly down-regulated in TNBC tissues with high expression of EGFR and related to the malignant phenotype of TNBC cells.** **A** The TNBC patients with EGFR level higher than the median value in the TCGA breast cancer database were selected. Their PDZK1 level gradually decreased as stage progressed. **P* < 0.05. **B** GSEA analysis results show that the low expression of PDZK1 were positively correlated with the activation of EGFR signaling pathway in TNBC patients with high EGFR level. **C**–**D** The TNBC patients with high expression of EGFR were divided into two groups according to the median *PDZK1* mRNA level. K-M curve results showed that the overall survival (C) and disease-free survival (D) of the group with lower PDZK1 levels were significantly shortened. **P* < 0.05.


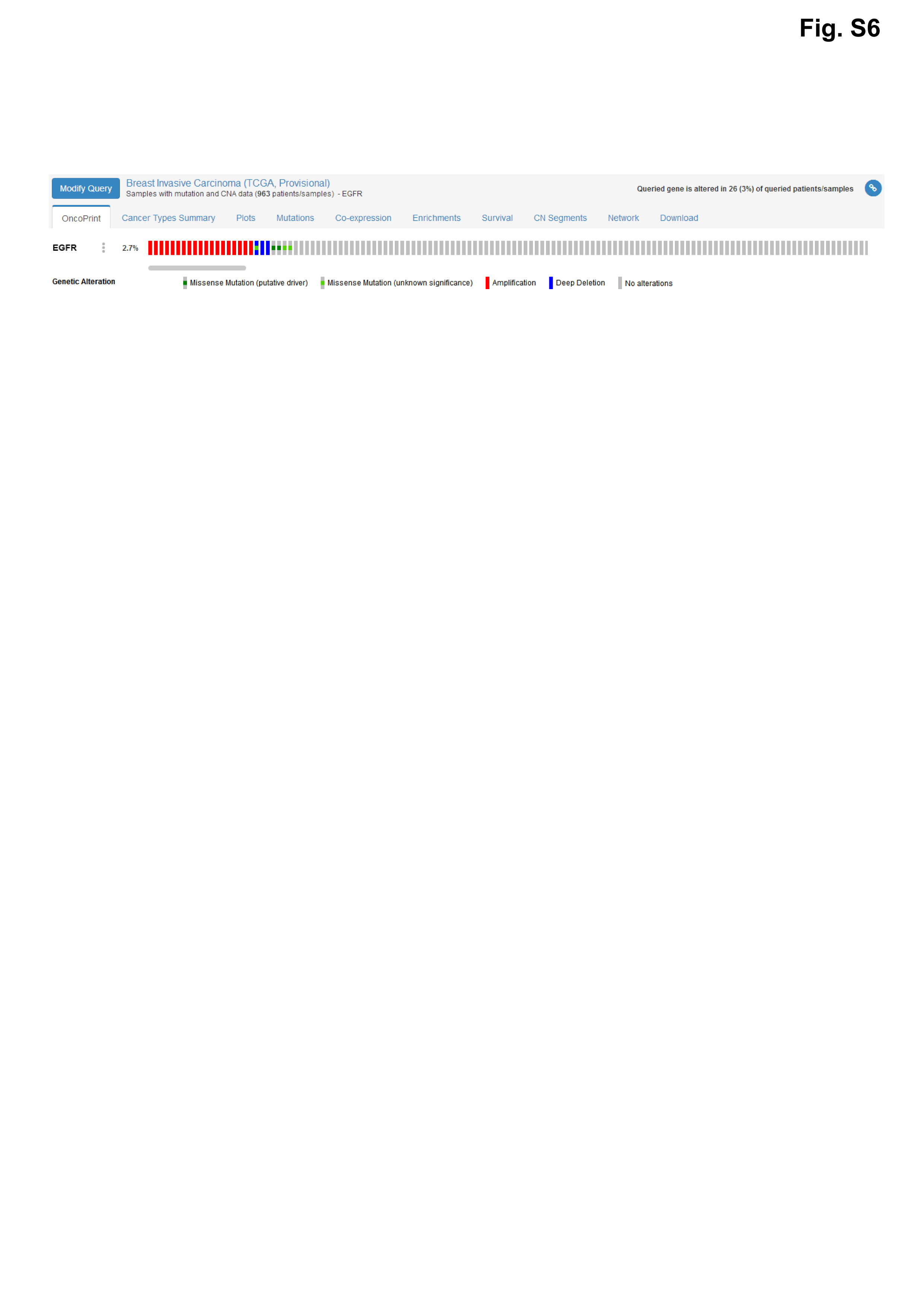


**Supplementary Fig. 7 TCGA cBioportal result revealed that *EGFR* gene was not mutated or amplified in TNBC tissues.**
