# Supplementary material for: PDZK1 suppresses TNBC development and sensitizes TNBC cells to erlotinib via the EGFR pathway
Source: Cell Death Dis. 2024 Apr 12;15(3):199. doi: 10.1038/s41419-024-06502-2 (PMC11009252; doi:10.1038/s41419-024-06502-2)
Supplement: Supplementary file 4 — Original Data File [file 41419_2024_6502_MOESM4_ESM.pdf]

Figure 1

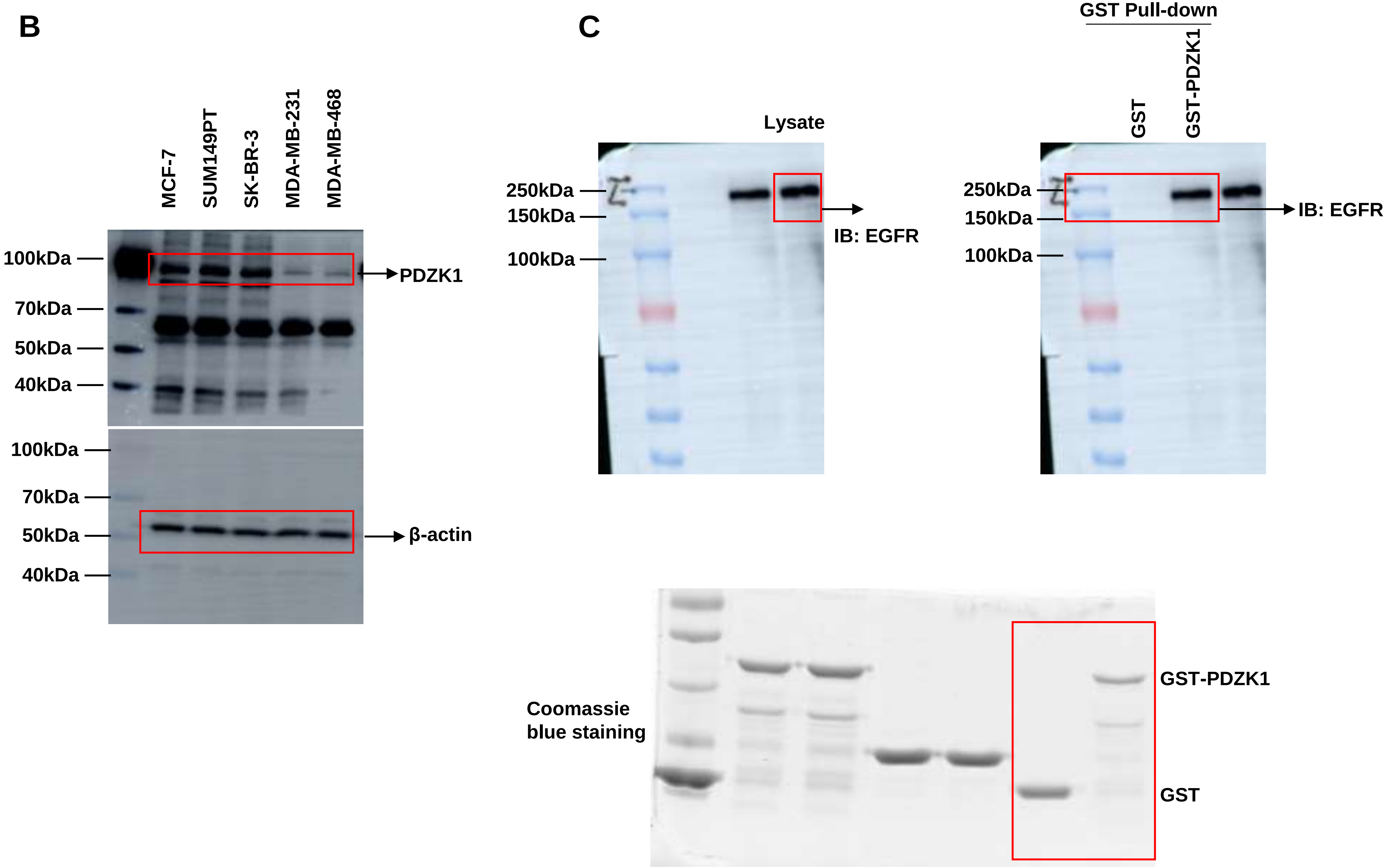
B
MCF-7
SUM149PT
SK-BR-3
MDA-MB-231
MDA-MB-468
100kDa
70kDa
50kDa
40kDa
PDZK1
100kDa
70kDa
50kDa
40kDa
β-actin
C
Lysate
GST Pull-down
GST
GST-PDZK1
250kDa
150kDa
100kDa
IB: EGFR
250kDa
150kDa
100kDa
IB: EGFR
Coomassie
blue staining
GST-PDZK1
GST

Figure 1

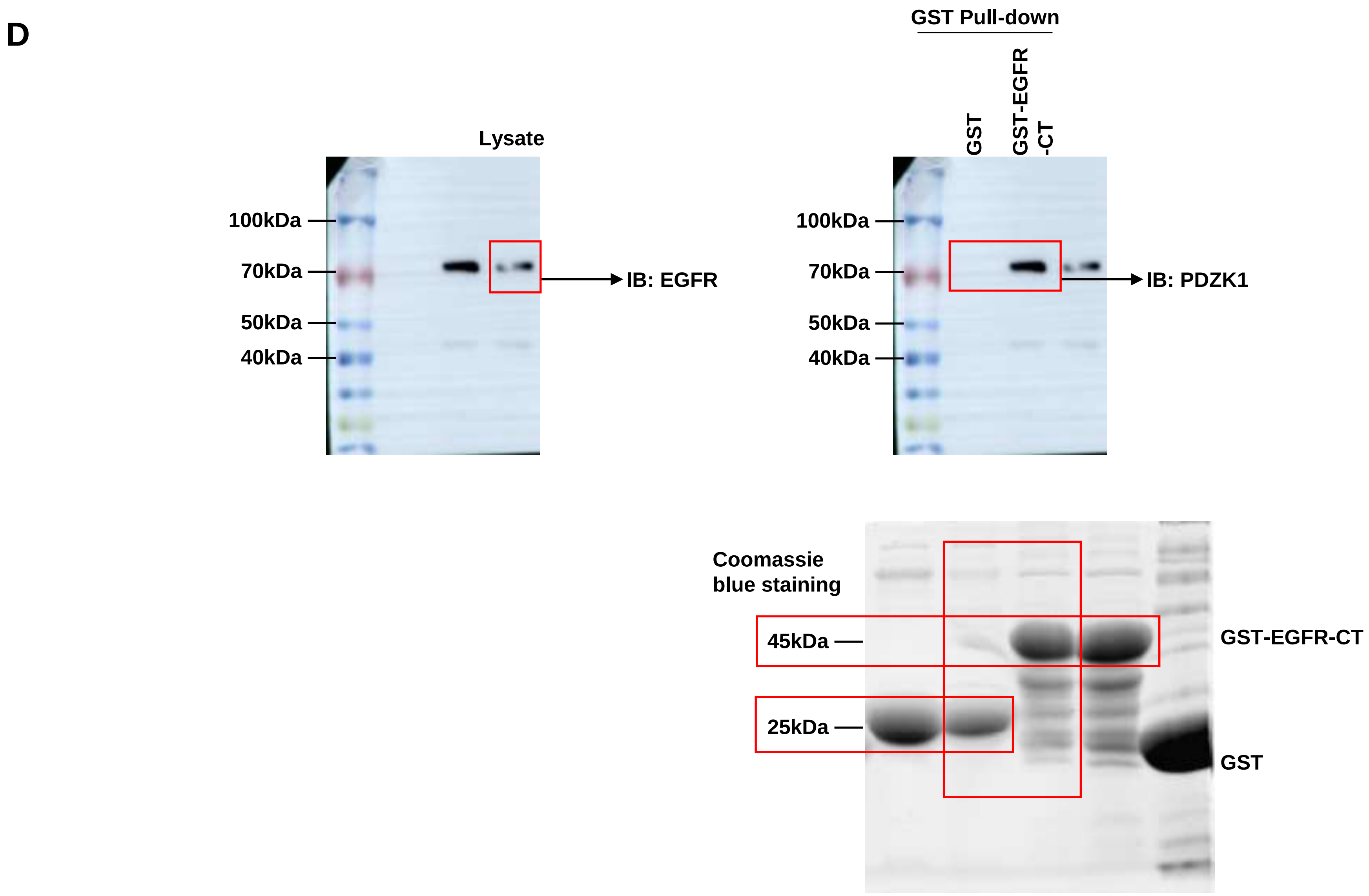
D
Lysate
100kDa
70kDa
50kDa
40kDa
IB: EGFR
GST Pull-down
GST
GST-EGFR
-CT
100kDa
70kDa
50kDa
40kDa
IB: PDZK1
Coomassie
blue staining
45kDa
25kDa
GST-EGFR-CT
GST

Figure 1

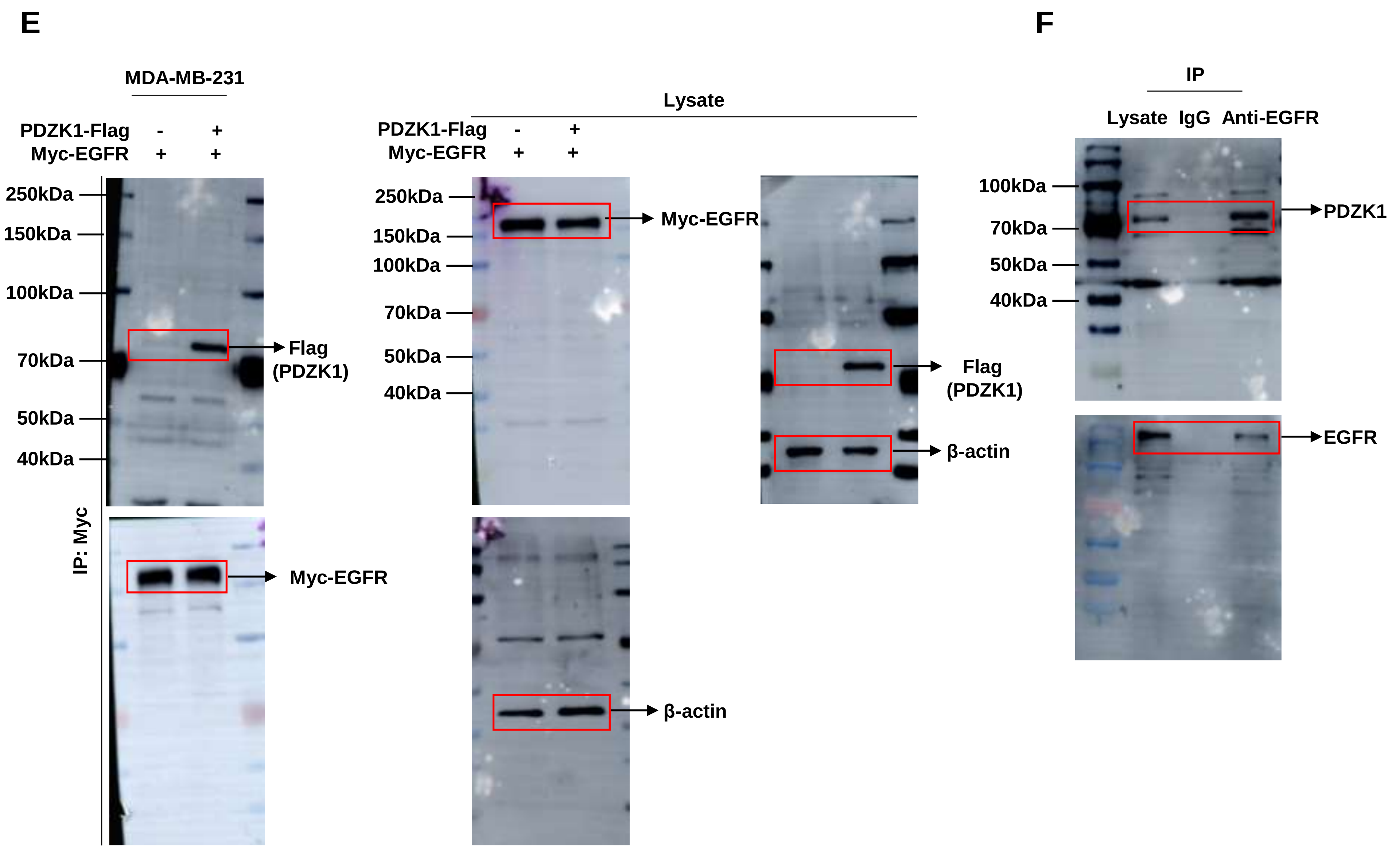
E
MDA-MB-231
PDZK1-Flag - +
Myc-EGFR + +
250kDa
150kDa
100kDa
70kDa
50kDa
40kDa
Flag (PDZK1)
IP: Myc
Myc-EGFR
Lysate
PDZK1-Flag - +
Myc-EGFR + +
250kDa
150kDa
100kDa
70kDa
50kDa
40kDa
Myc-EGFR
Flag (PDZK1)
β-actin
β-actin
F
IP
Lysate IgG Anti-EGFR
100kDa
70kDa
50kDa
40kDa
PDZK1
EGFR

Figure 1

G

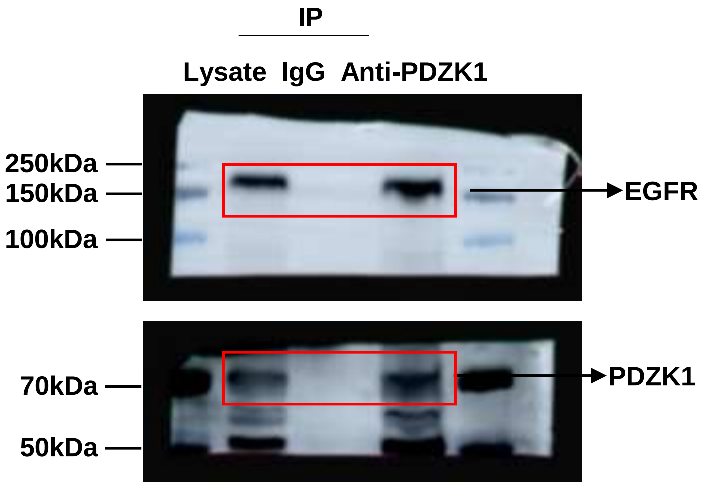
IP
Lysate
IgG
Anti-PDZK1
250kDa
150kDa
100kDa
EGFR
70kDa
50kDa
PDZK1

Figure 1

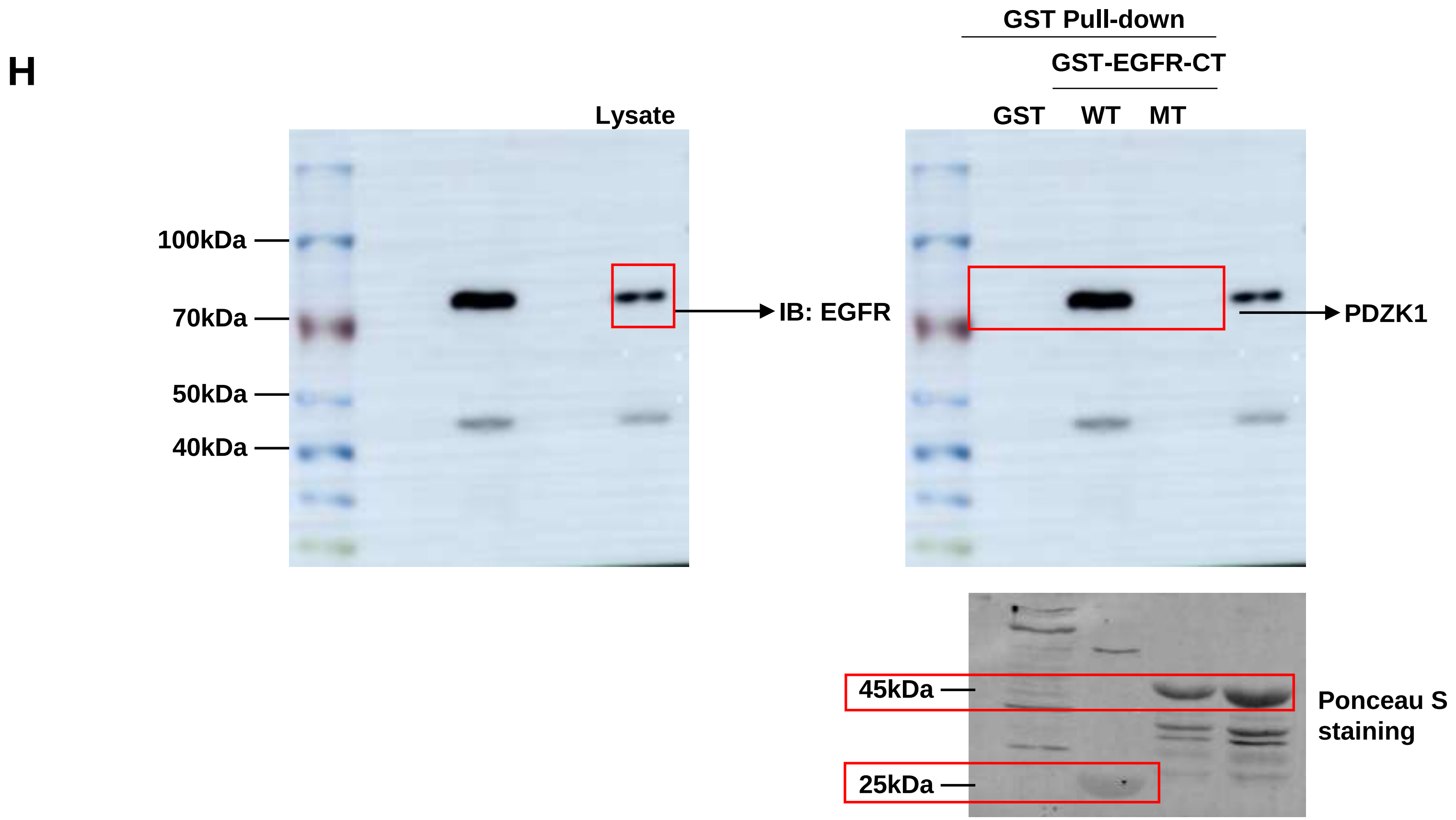
H
GST Pull-down
GST-EGFR-CT
Lysate
GST
WT
MT
100kDa
70kDa
50kDa
40kDa
IB: EGFR
PDZK1
45kDa
25kDa
Ponceau S
staining

Figure 1

I

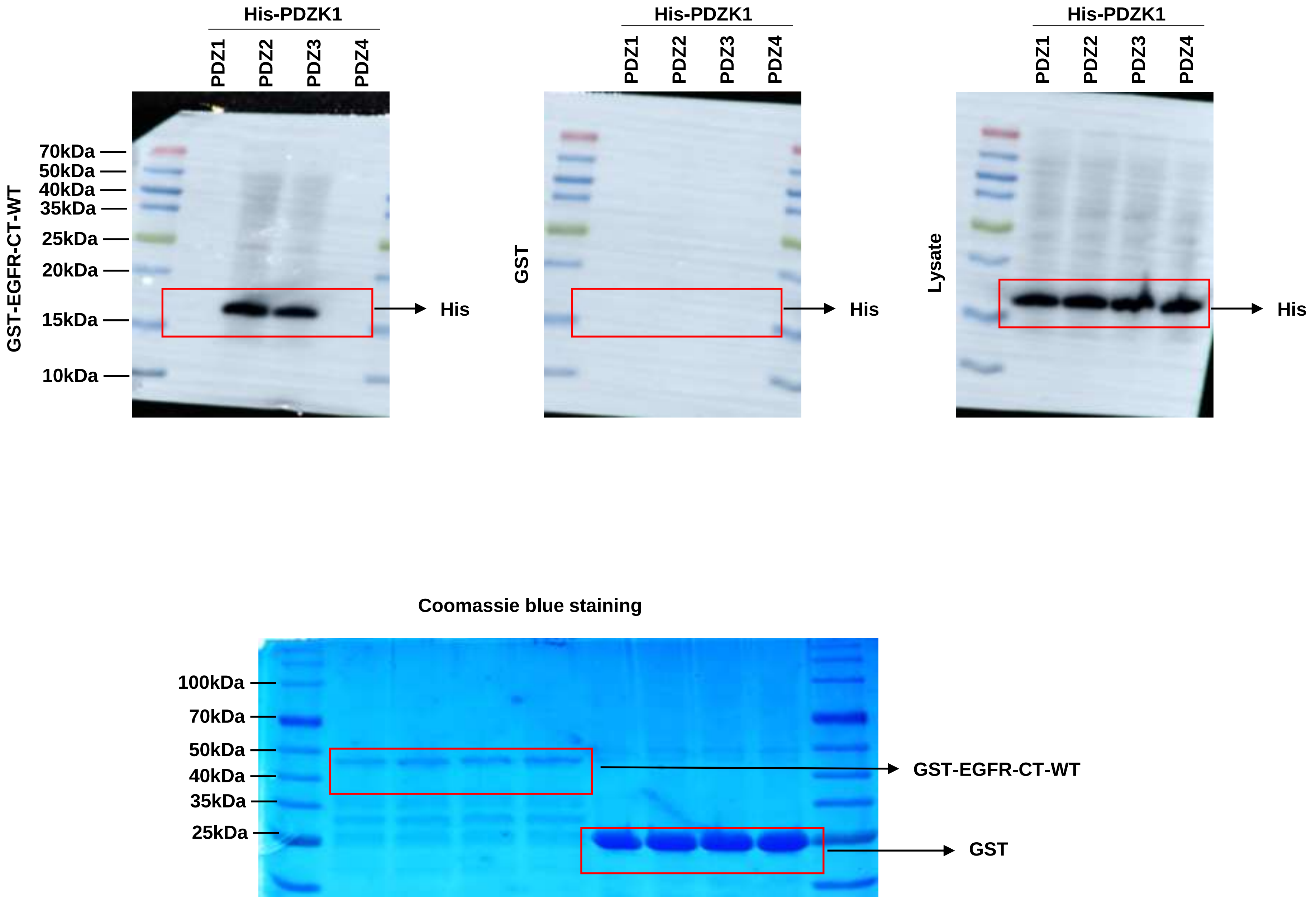
His-PDZK1
PDZ1
PDZ2
PDZ3
PDZ4
70kDa
50kDa
40kDa
35kDa
25kDa
20kDa
15kDa
10kDa
GST-EGFR-CT-WT
His
GST
Lysate
Coomassie blue staining
100kDa
GST-EGFR-CT-WT

Figure 1

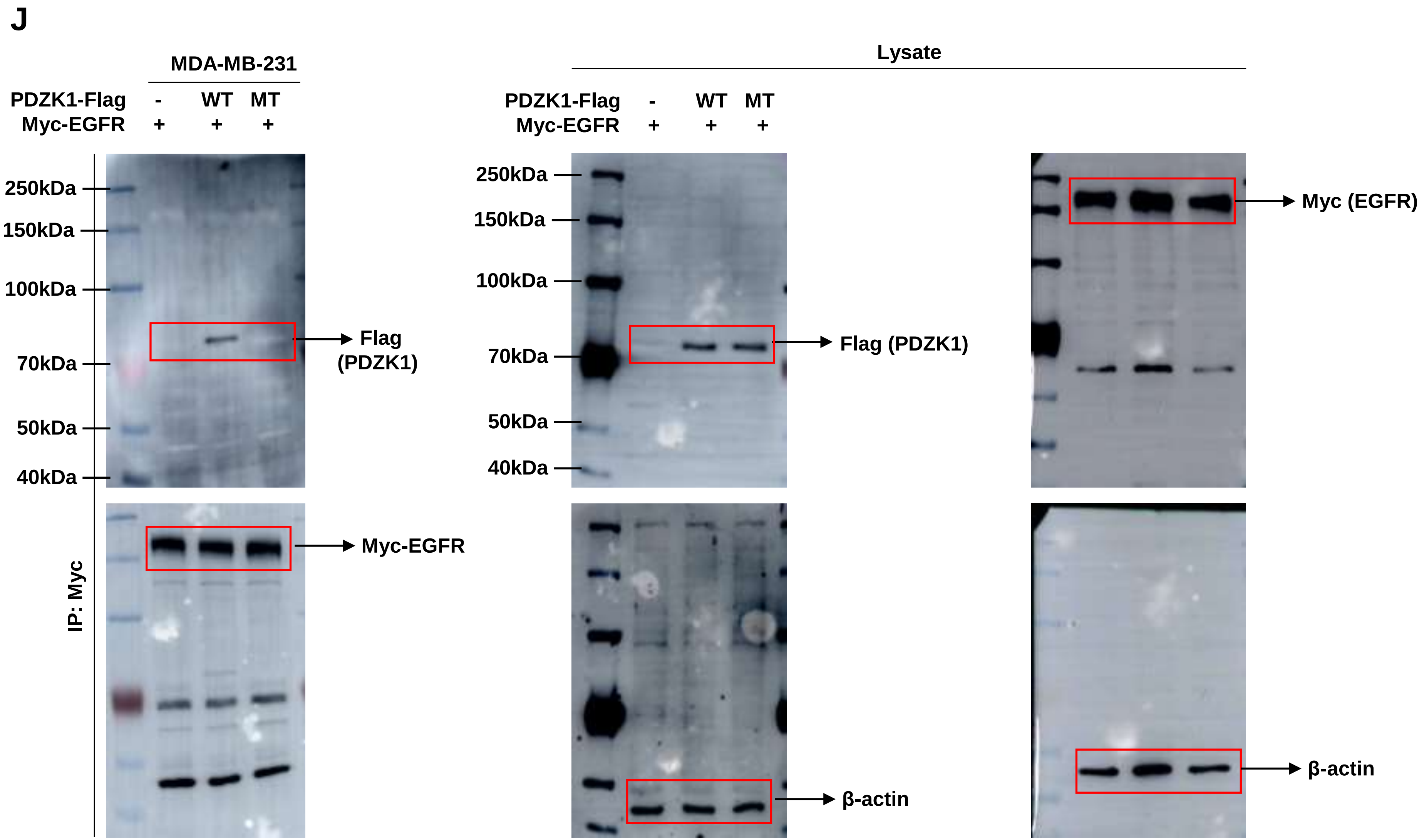

J
MDA-MB-231
Lysate
PDZK1-Flag - WT MT
Myc-EGFR + + +
PDZK1-Flag - WT MT
Myc-EGFR + + +
250kDa
150kDa
100kDa
70kDa
50kDa
40kDa
Flag (PDZK1)
IP: Myc
Myc-EGFR
250kDa
150kDa
100kDa
70kDa
50kDa
40kDa
Flag (PDZK1)
β-actin
Myc (EGFR)
β-actin

Figure 2

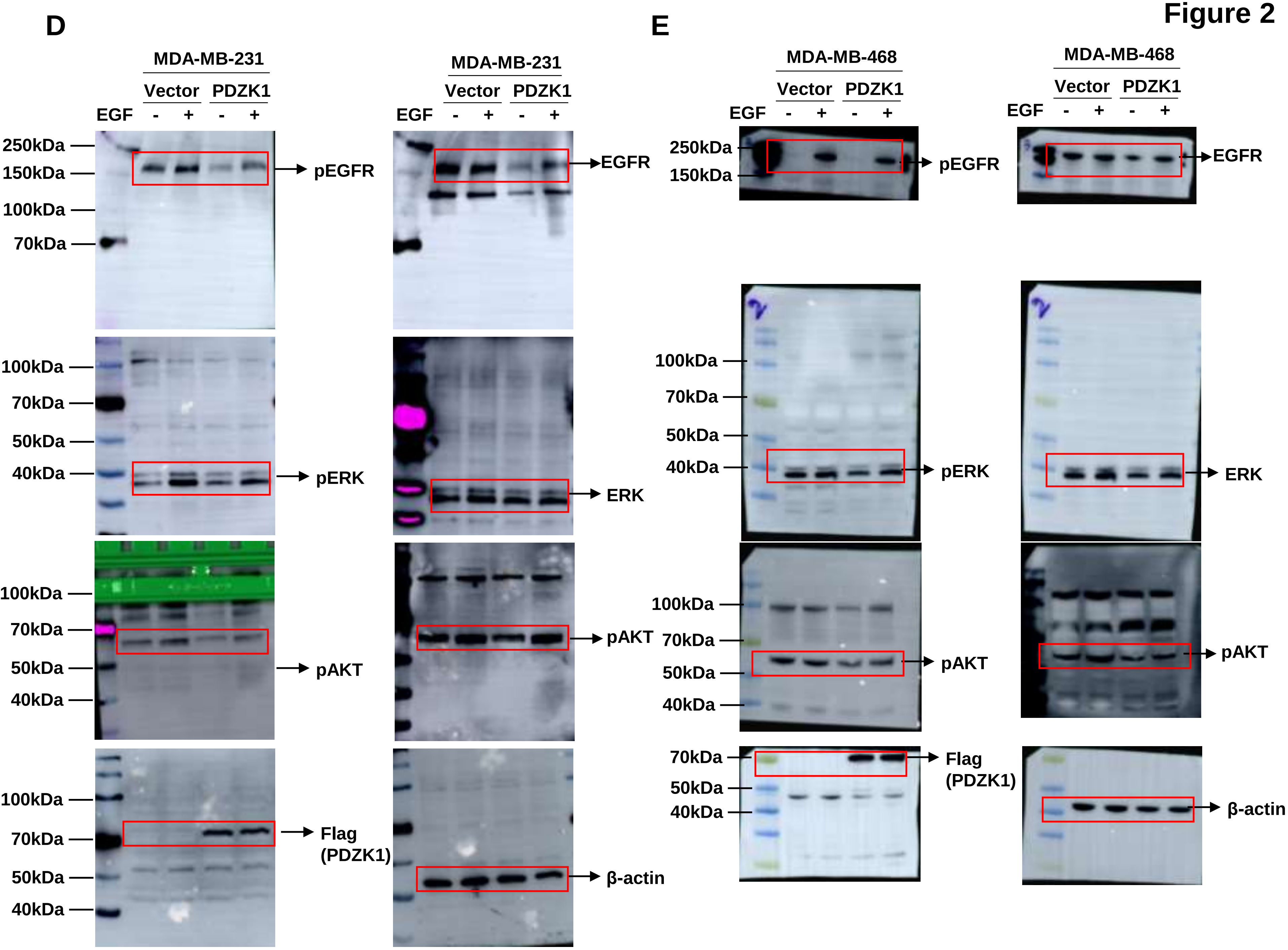

D
MDA-MB-231
Vector
PDZK1
EGF - + - +
250kDa
150kDa
100kDa
70kDa
pEGFR
EGFR
50kDa
40kDa
pERK
ERK
pAKT
Flag (PDZK1)
β-actin
E
MDA-MB-468

Figure 2

F

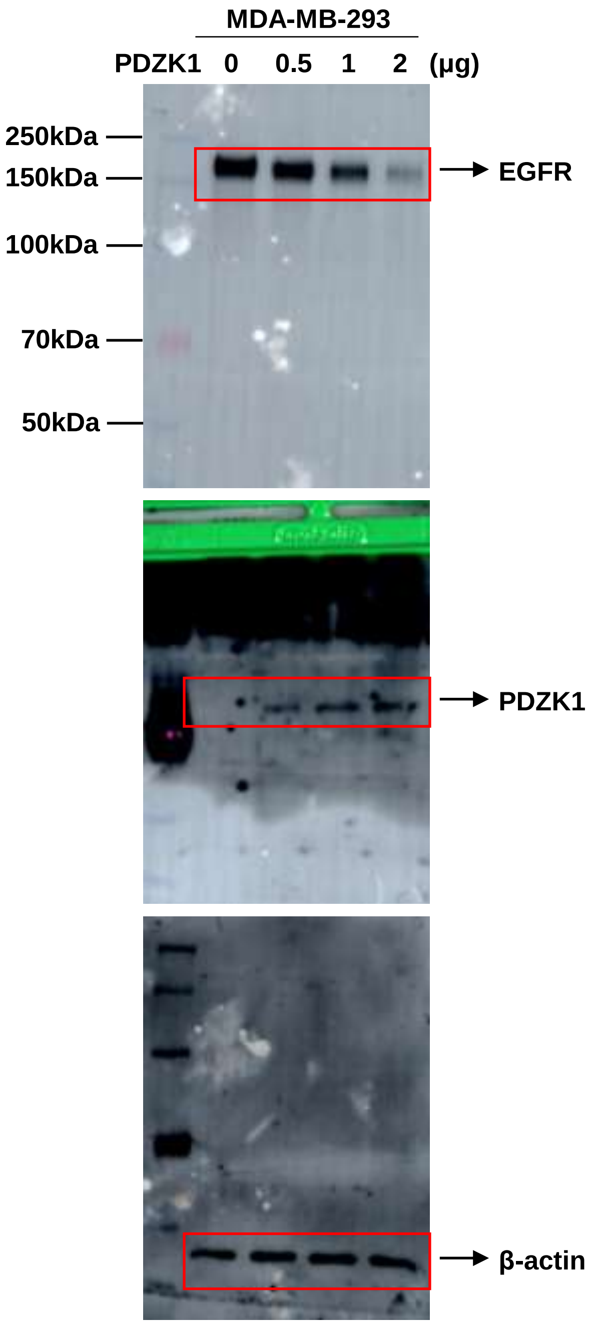
MDA-MB-293
PDZK1 0 0.5 1 2 (μg)
250kDa
150kDa
100kDa
70kDa
50kDa
EGFR
PDZK1
β-actin

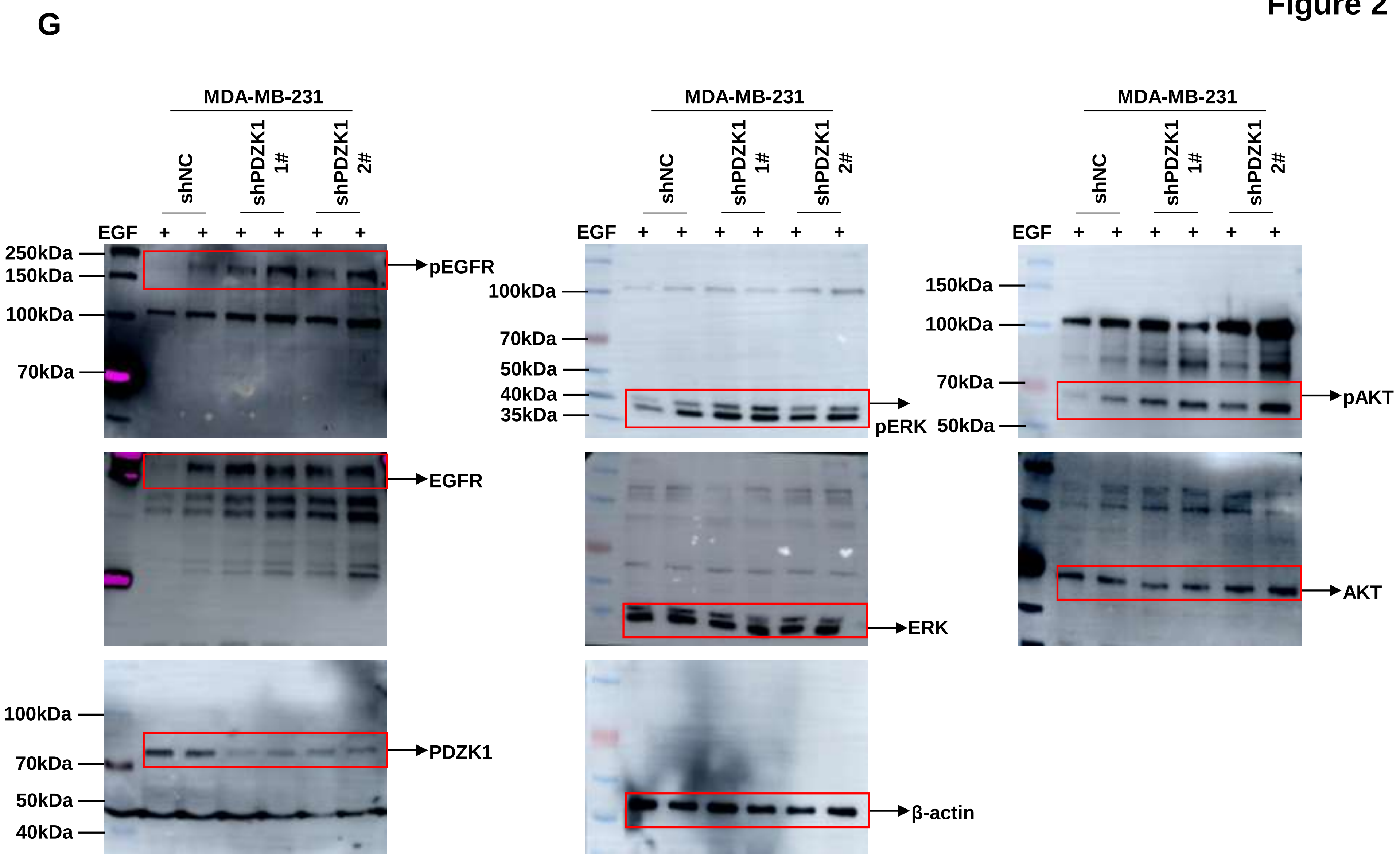
G
MDA-MB-231
shNC
shPDZK1 1#
shPDZK1 2#
EGF + + + + + +
250kDa
150kDa
100kDa
70kDa
pEGFR
EGFR
PDZK1
50kDa
40kDa
35kDa
pERK
ERK
β-actin
pAKT
AKT

Figure 2

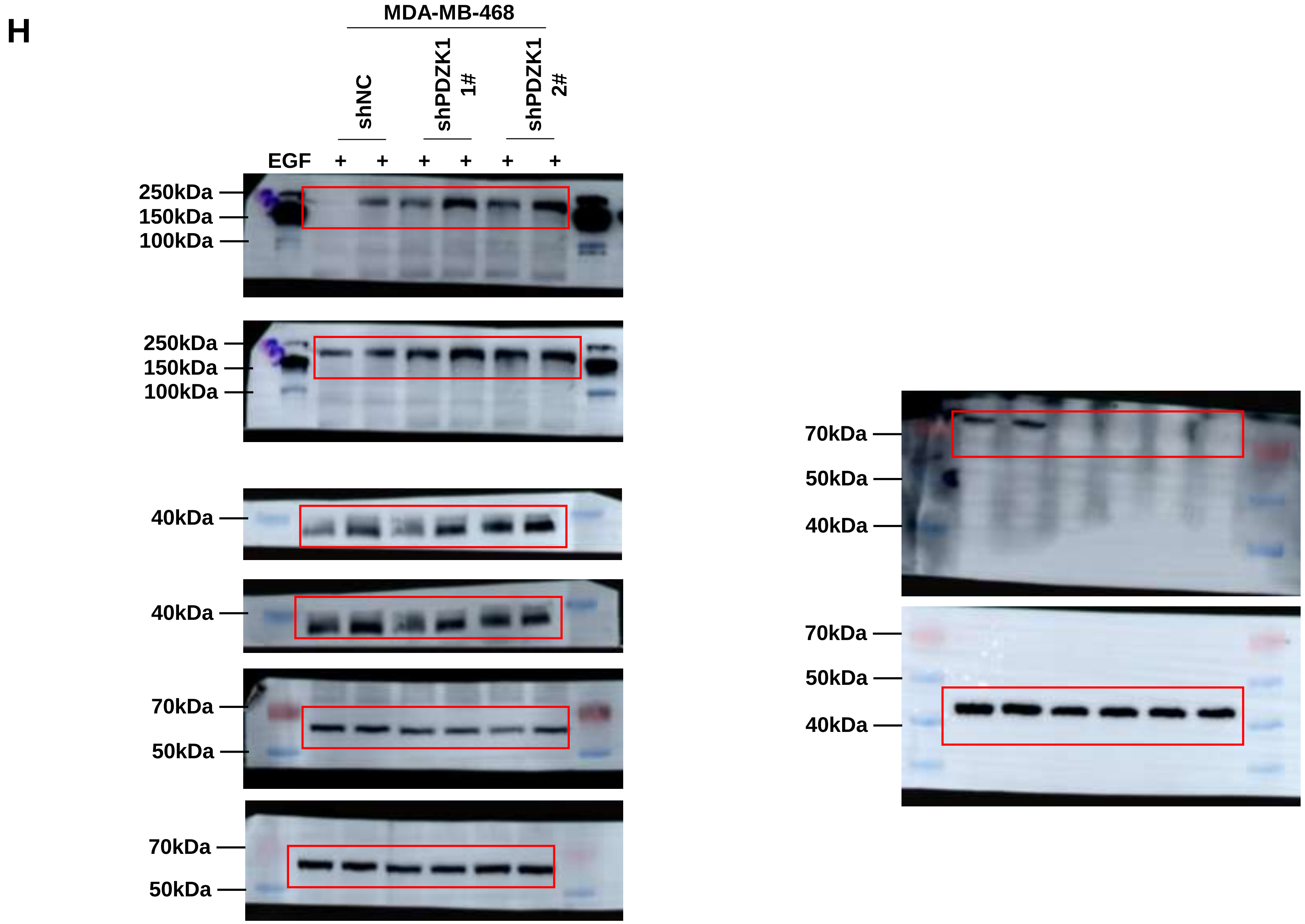
H
MDA-MB-468
shNC
shPDZK1 1#
shPDZK1 2#
EGF + + + + + +
250kDa
150kDa
100kDa
250kDa
150kDa
100kDa
40kDa
40kDa
70kDa
50kDa
70kDa
50kDa
70kDa
50kDa
40kDa
70kDa
50kDa
40kDa

Figure 2

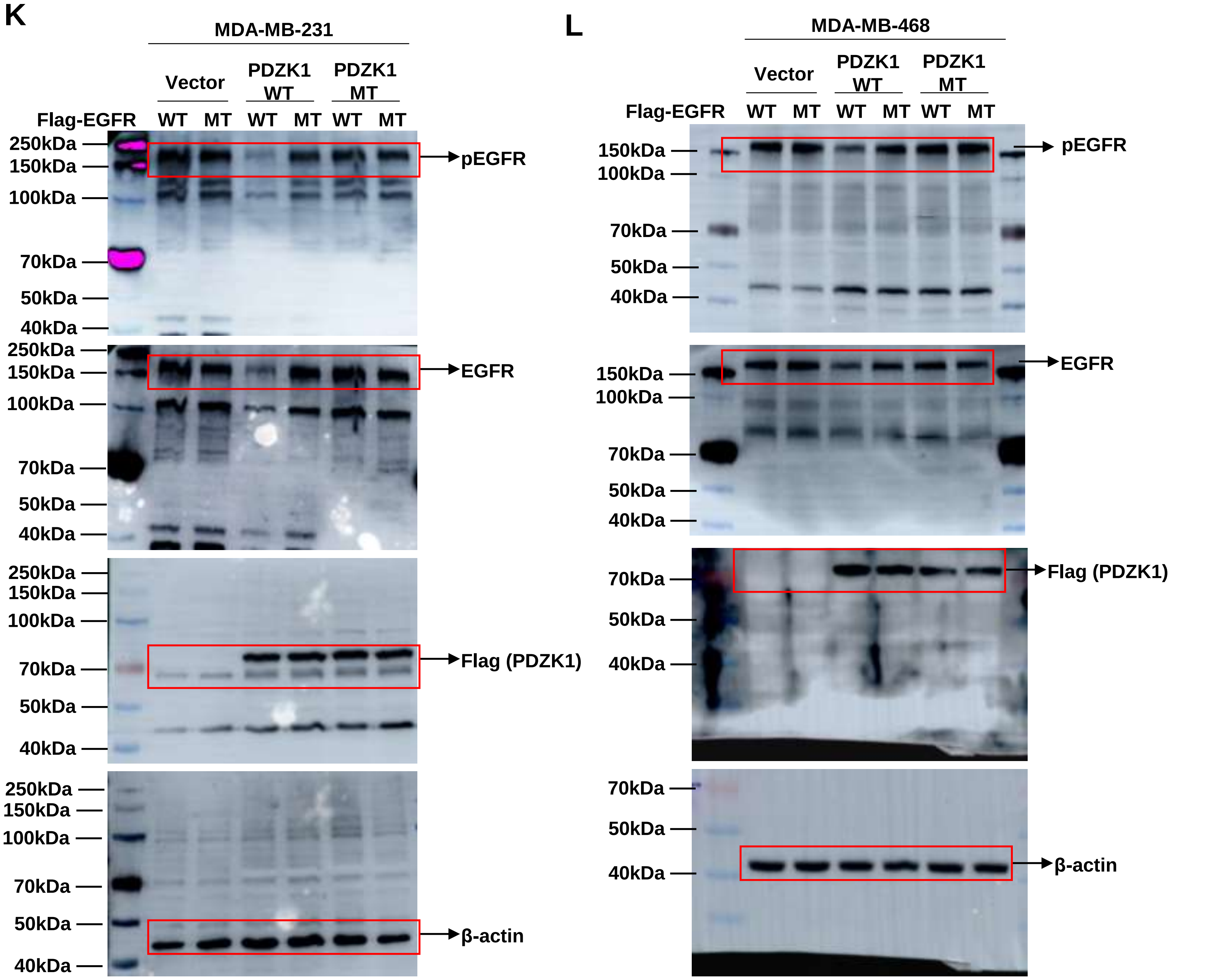

K
MDA-MB-231
Vector
PDZK1 WT
PDZK1 MT
Flag-EGFR
WT
MT
WT
MT
WT
MT
250kDa
150kDa
100kDa
70kDa
50kDa
40kDa
pEGFR
EGFR
Flag (PDZK1)
β-actin
L
MDA-MB-468
Vector
PDZK1 WT
PDZK1 MT
Flag-EGFR
WT
MT
WT
MT
WT
MT
150kDa
100kDa
70kDa
50kDa
40kDa
pEGFR
EGFR
Flag (PDZK1)
β-actin

Figure 3

A

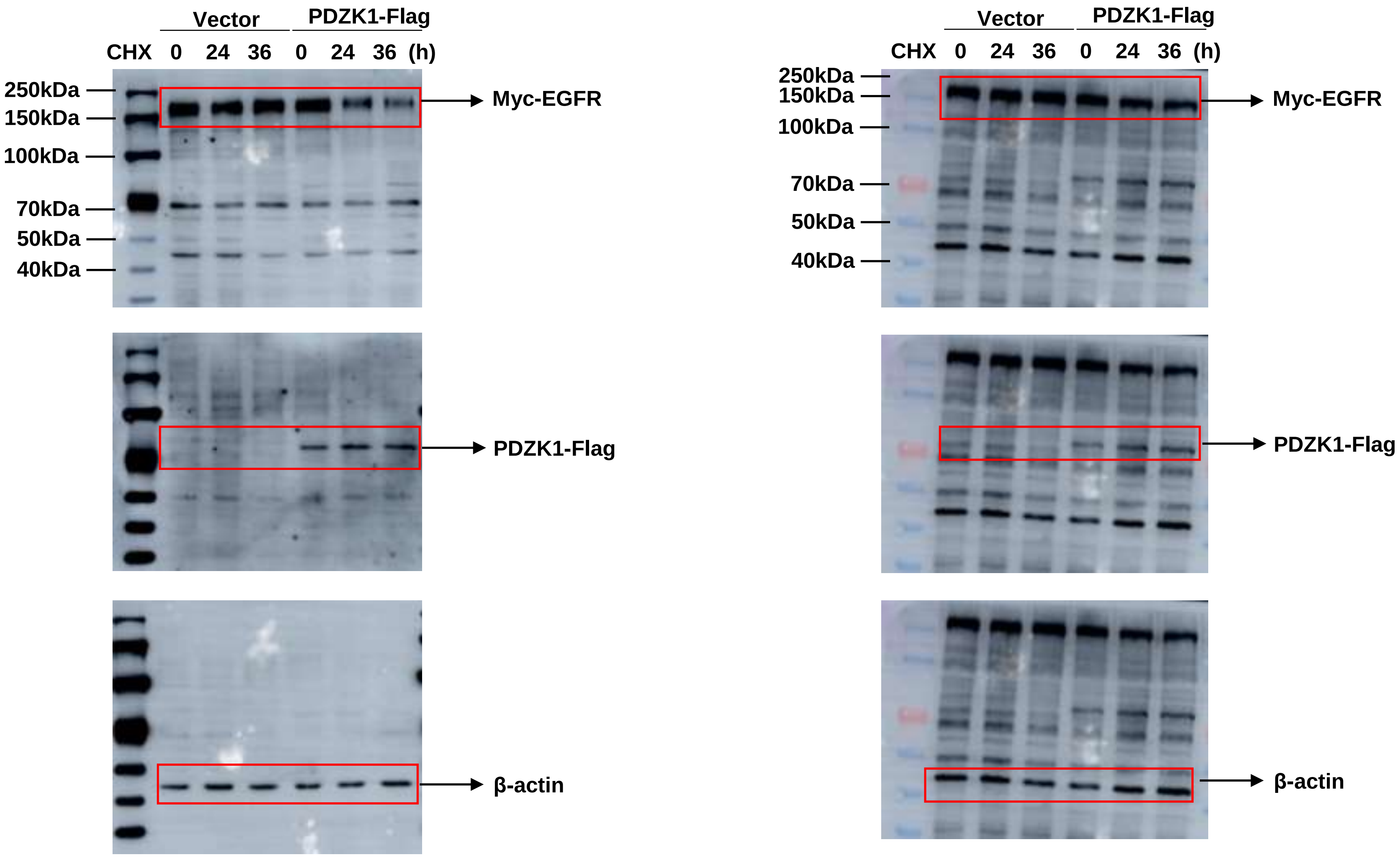
Vector
PDZK1-Flag
CHX 0 24 36 0 24 36 (h)
250kDa
150kDa
100kDa
70kDa
50kDa
40kDa
Myc-EGFR
PDZK1-Flag
β-actin
Vector
PDZK1-Flag
CHX 0 24 36 0 24 36 (h)
250kDa
150kDa
100kDa
70kDa
50kDa
40kDa
Myc-EGFR
PDZK1-Flag
β-actin

Figure 3

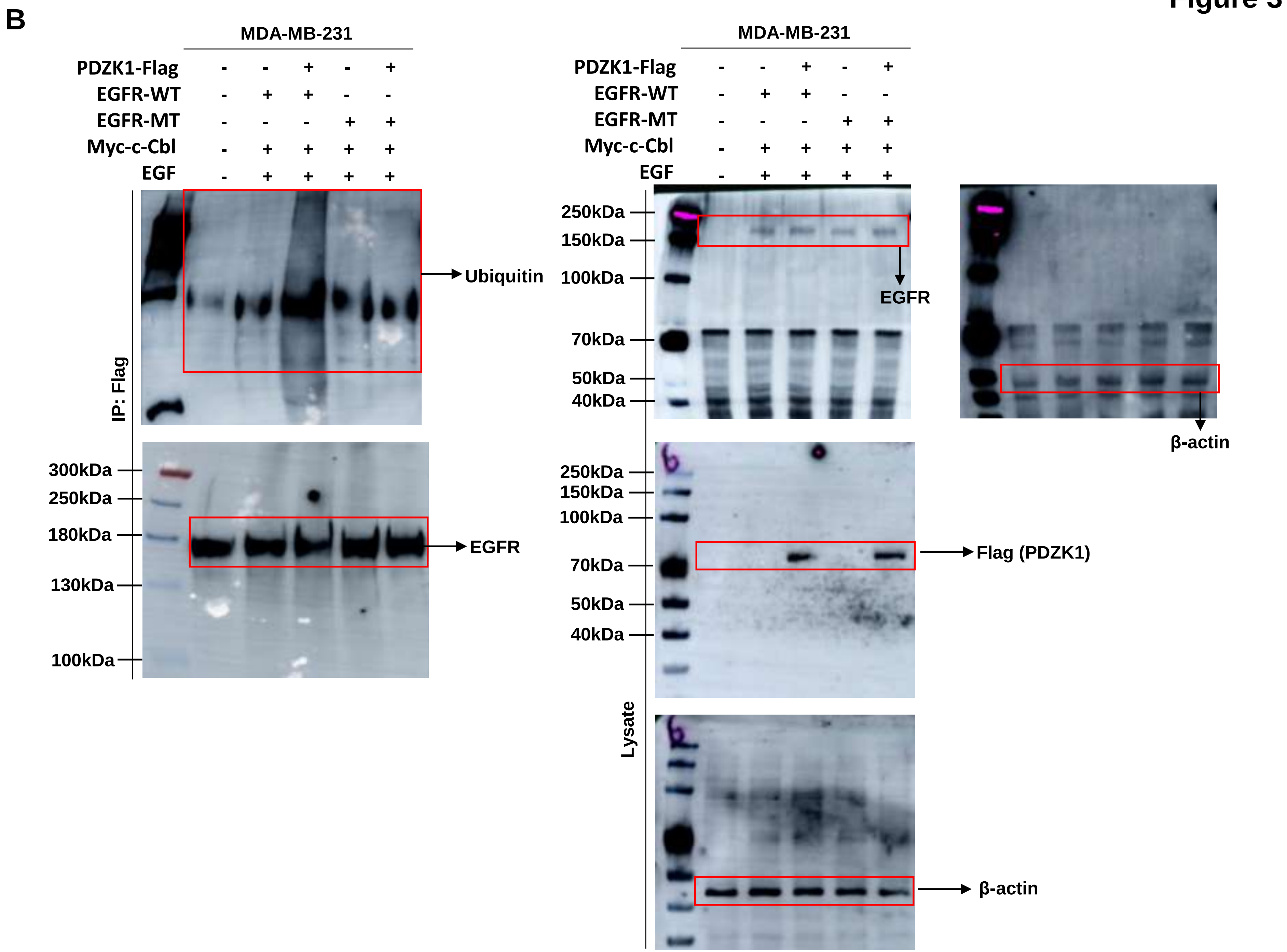

B
MDA-MB-231
PDZK1-Flag - - + - +
EGFR-WT - + + - -
EGFR-MT - - - + +
Myc-c-Cbl - + + + +
EGF - + + + +
Ubiquitin
IP: Flag
300kDa
250kDa
180kDa
130kDa
100kDa
EGFR
MDA-MB-231
PDZK1-Flag - - + - +
EGFR-WT - + + - -
EGFR-MT - - - + +
Myc-c-Cbl - + + + +
EGF - + + + +
250kDa
150kDa
100kDa
70kDa
50kDa
40kDa
EGFR
β-actin
Flag (PDZK1)
Lysate
β-actin

Figure 3

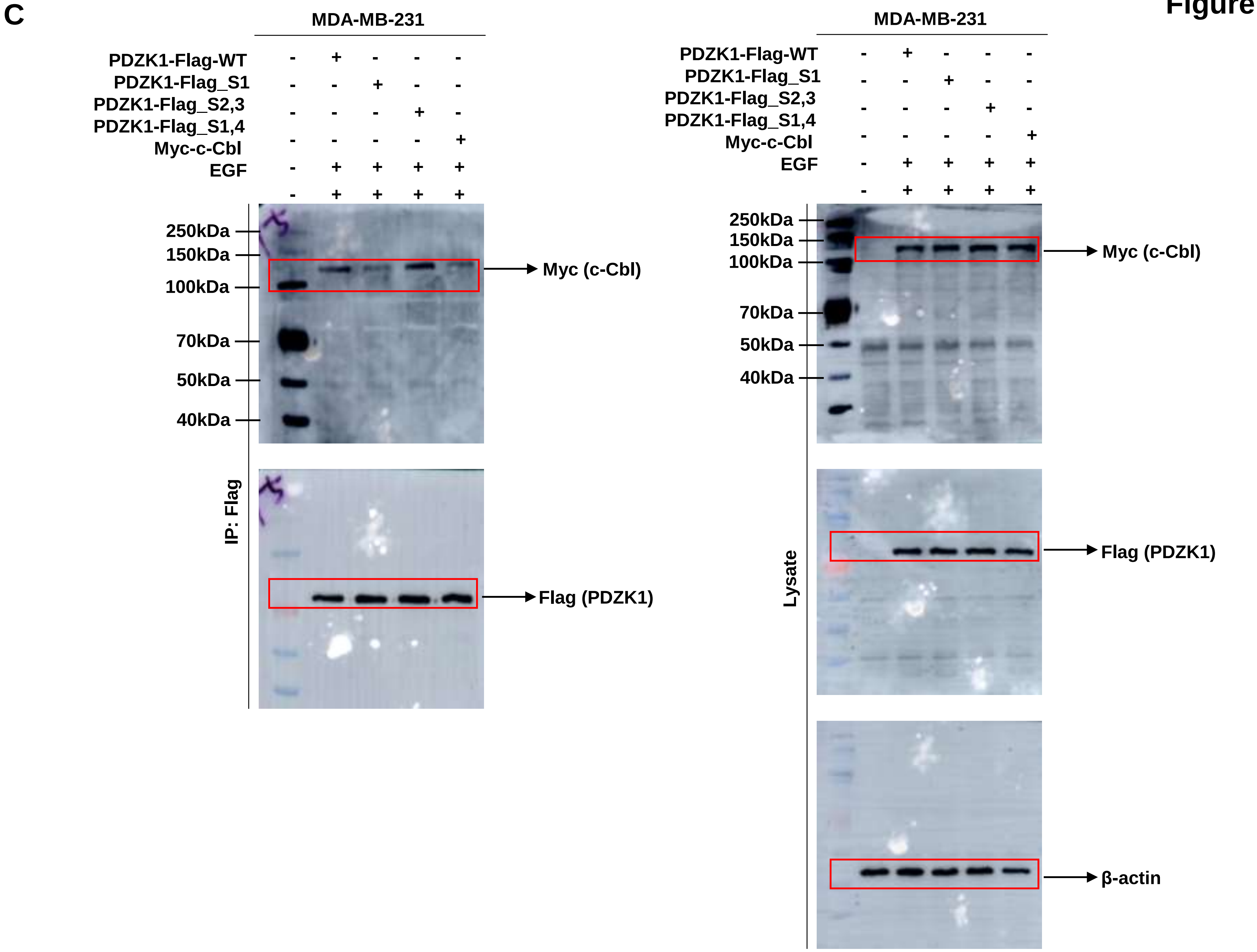
C
MDA-MB-231
PDZK1-Flag-WT
PDZK1-Flag_S1
PDZK1-Flag_S2,3
PDZK1-Flag_S1,4
Myc-c-Cbl
EGF
250kDa
150kDa
100kDa
70kDa
50kDa
40kDa
Myc (c-Cbl)
Flag (PDZK1)
IP: Flag
MDA-MB-231
PDZK1-Flag-WT
PDZK1-Flag_S1
PDZK1-Flag_S2,3
PDZK1-Flag_S1,4
Myc-c-Cbl
EGF
250kDa
150kDa
100kDa
70kDa
50kDa
40kDa
Myc (c-Cbl)
Flag (PDZK1)
β-actin
Lysate

Figure 3

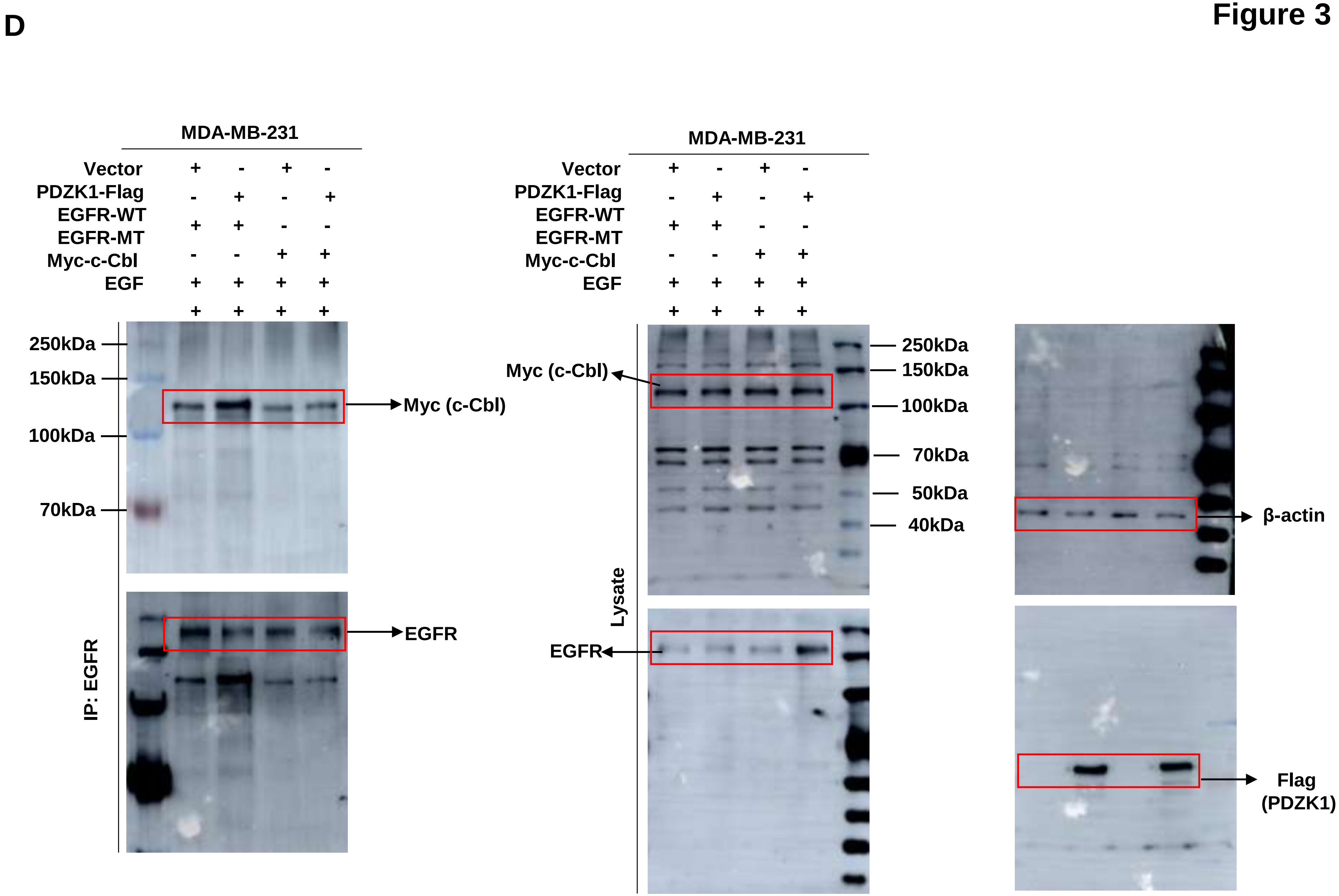
D
MDA-MB-231
Vector + - + -
PDZK1-Flag - + - +
EGFR-WT + + - -
EGFR-MT - - + +
Myc-c-Cbl
EGF + + + +
+ + + +
250kDa
150kDa
100kDa
70kDa
Myc (c-Cbl)
EGFR
IP: EGFR
MDA-MB-231
Vector + - + -
PDZK1-Flag - + - +
EGFR-WT + + - -
EGFR-MT - - + +
Myc-c-Cbl
EGF + + + +
+ + + +
Myc (c-Cbl)
250kDa
150kDa
100kDa
70kDa
50kDa
40kDa
β-actin
Lysate
EGFR
Flag (PDZK1)

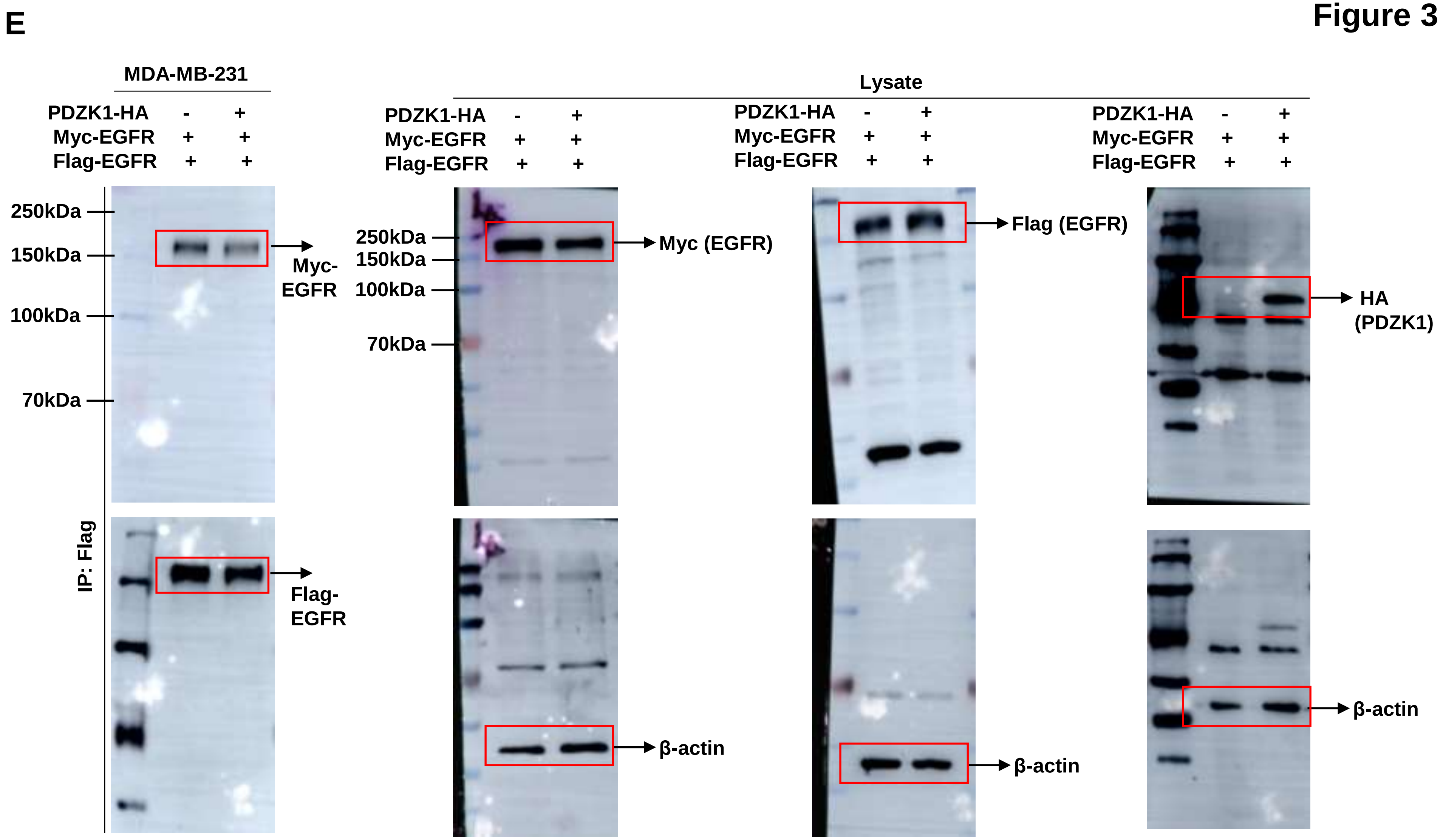
E
MDA-MB-231
Lysate
PDZK1-HA - +
Myc-EGFR + +
Flag-EGFR + +
250kDa
150kDa
100kDa
70kDa
IP: Flag
Myc-EGFR
Flag-EGFR
Myc (EGFR)
Flag (EGFR)
HA (PDZK1)
β-actin

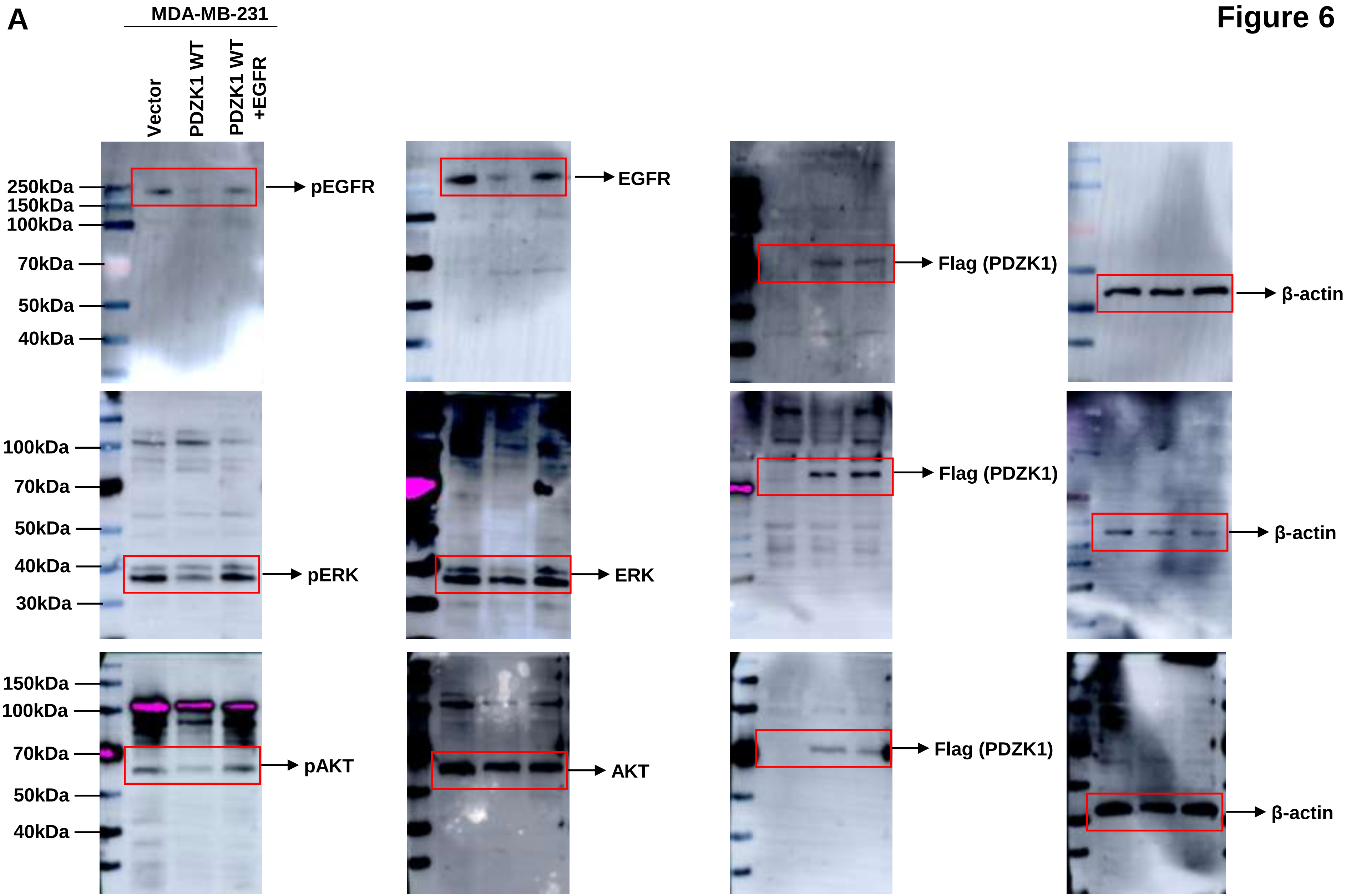
A
MDA-MB-231
Vector
PDZK1 WT
PDZK1 WT +EGFR
250kDa
150kDa
100kDa
70kDa
50kDa
40kDa
pEGFR
EGFR
Flag (PDZK1)
β-actin
100kDa
70kDa
50kDa
40kDa
30kDa
pERK
ERK
Flag (PDZK1)
β-actin
150kDa
100kDa
70kDa
50kDa
40kDa
pAKT
AKT
Flag (PDZK1)
β-actin

Figure 6

Figure 6

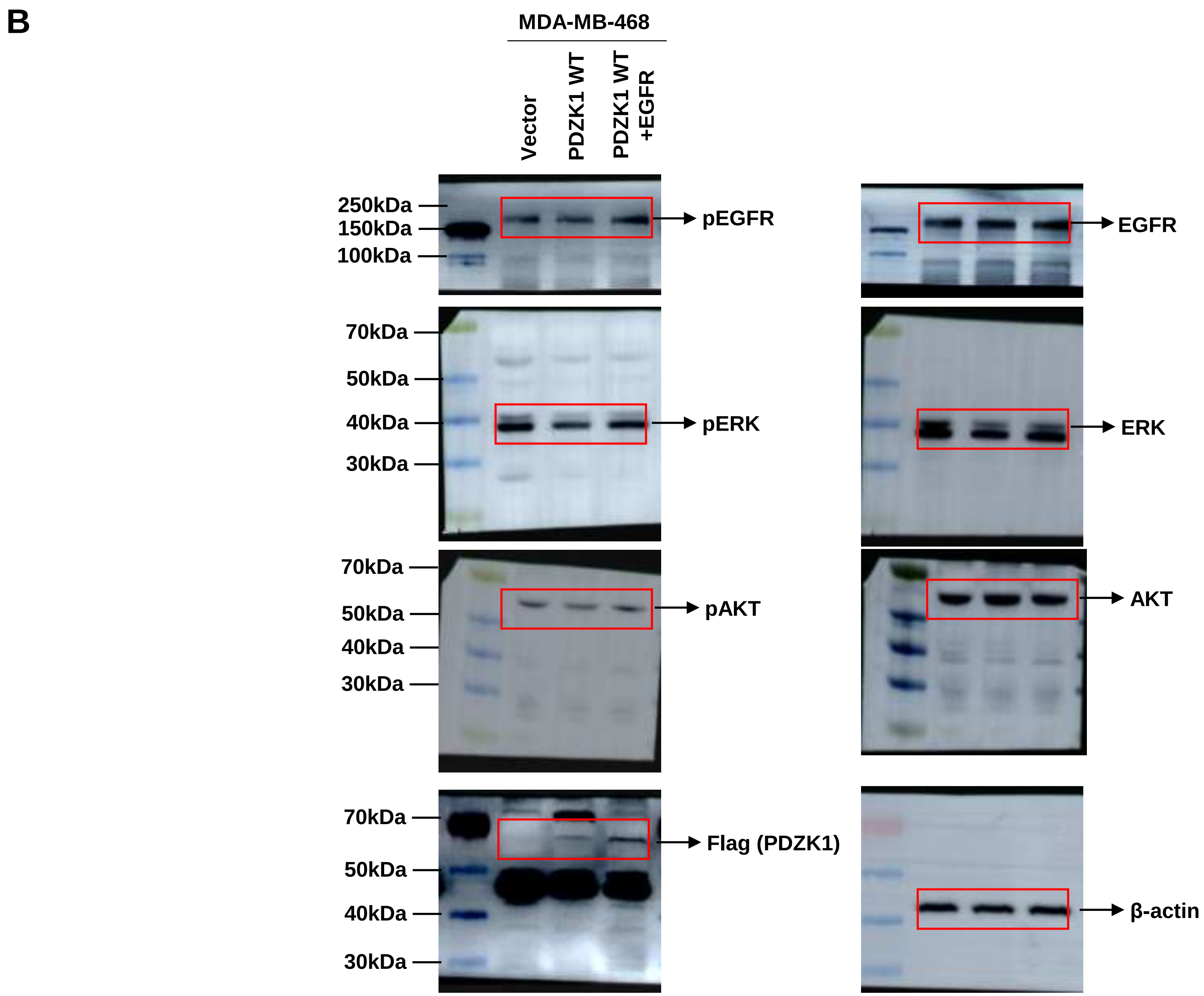

B
MDA-MB-468
Vector
PDZK1 WT
PDZK1 WT +EGFR
250kDa
150kDa
100kDa
pEGFR
EGFR
70kDa
50kDa
40kDa
30kDa
pERK
ERK
70kDa
50kDa
40kDa
30kDa
pAKT
AKT
70kDa
50kDa
40kDa
30kDa
Flag (PDZK1)
β-actin

Figure 6

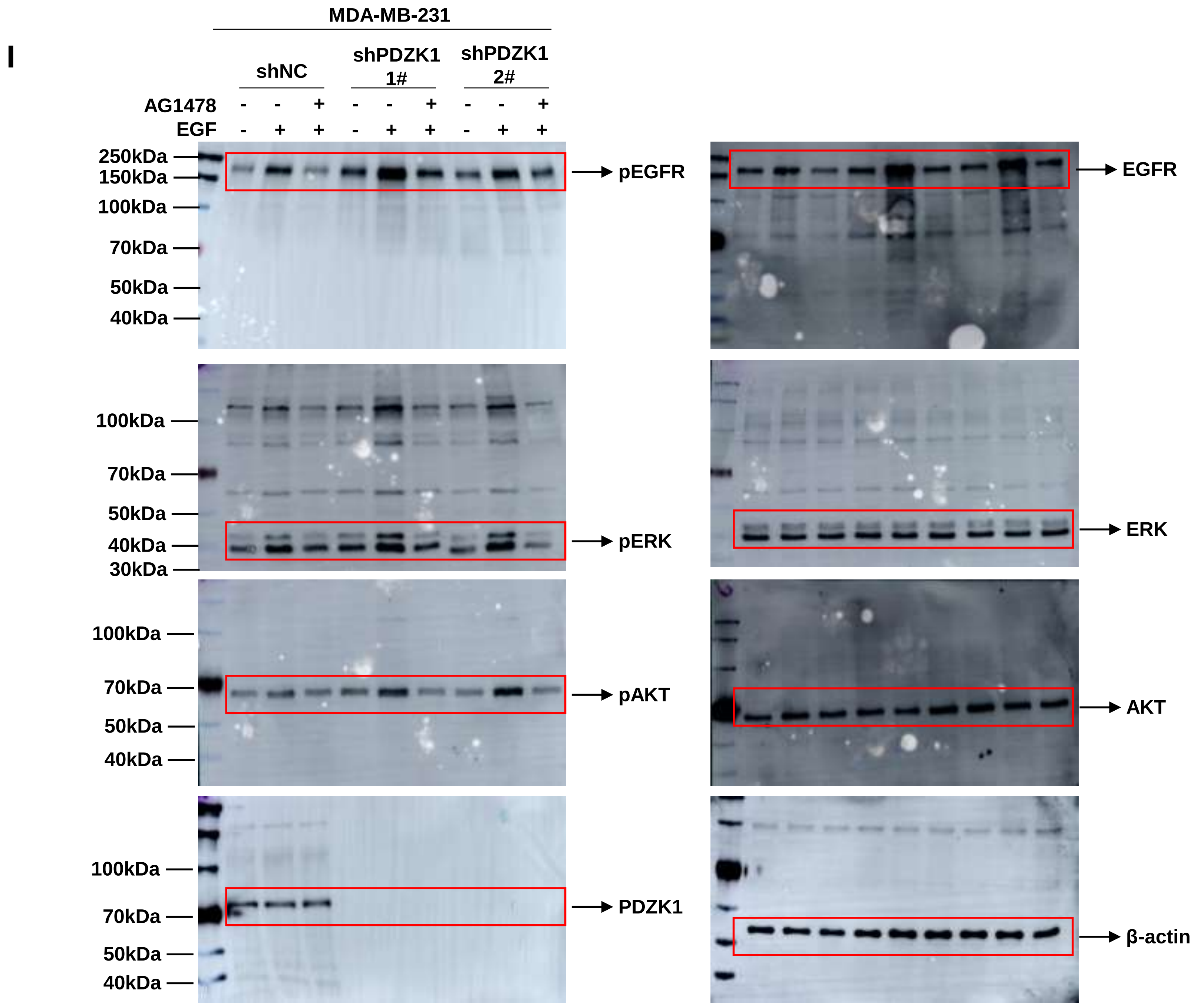

I
MDA-MB-231
shNC
shPDZK1 1#
shPDZK1 2#
AG1478 - - + - - + - - +
EGF - + + - + + - + +
250kDa
150kDa
100kDa
70kDa
50kDa
40kDa
30kDa
pEGFR
EGFR
pERK
ERK
pAKT
AKT
PDZK1
β-actin

Figure 6

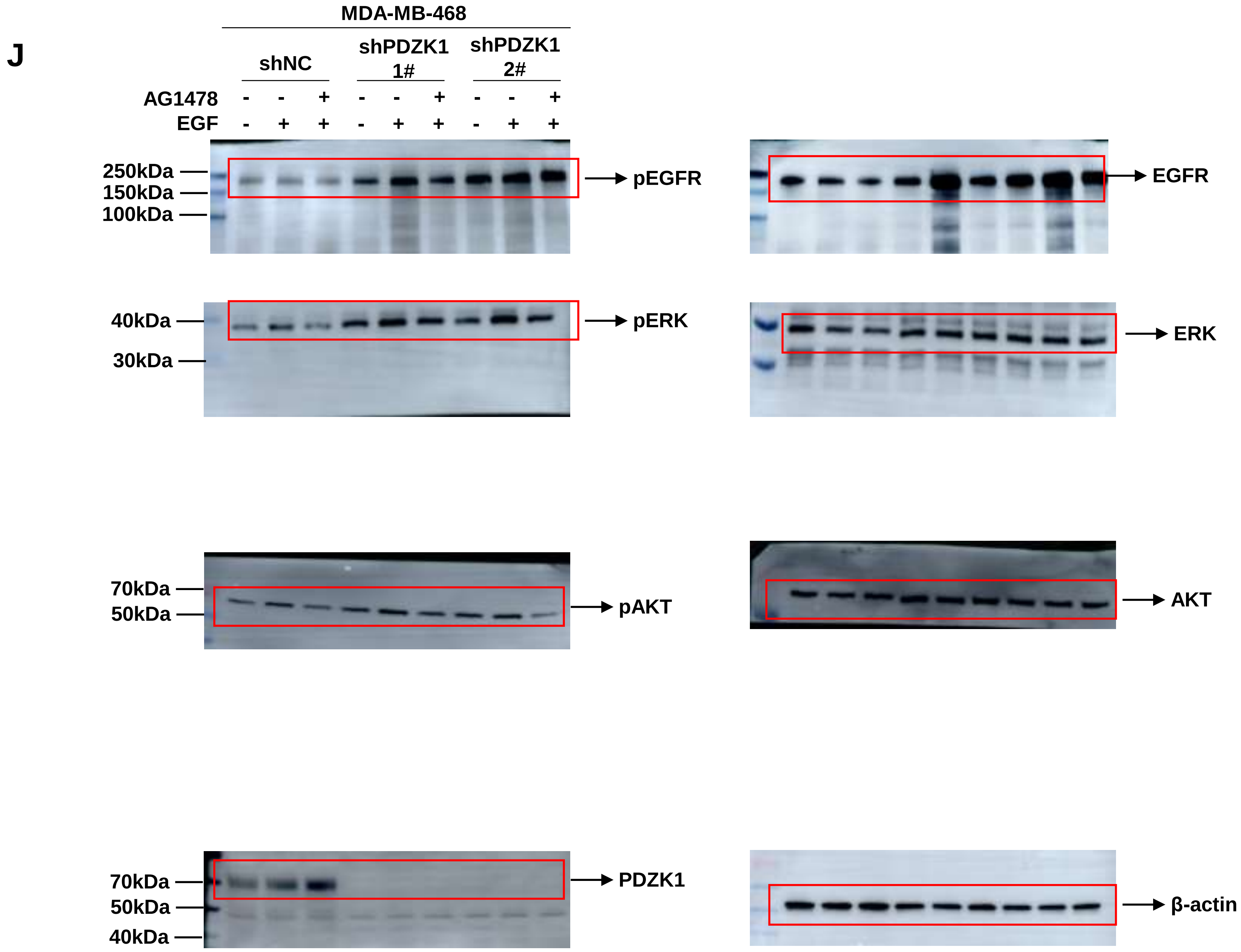
J
MDA-MB-468
shNC
shPDZK1 1#
shPDZK1 2#
AG1478 - - + - - + - - +
EGF - + + - + + - + +
250kDa
150kDa
100kDa
pEGFR
EGFR
40kDa
30kDa
pERK
ERK
70kDa
50kDa
pAKT
AKT
70kDa
50kDa
40kDa
PDZK1
β-actin

Figure 7

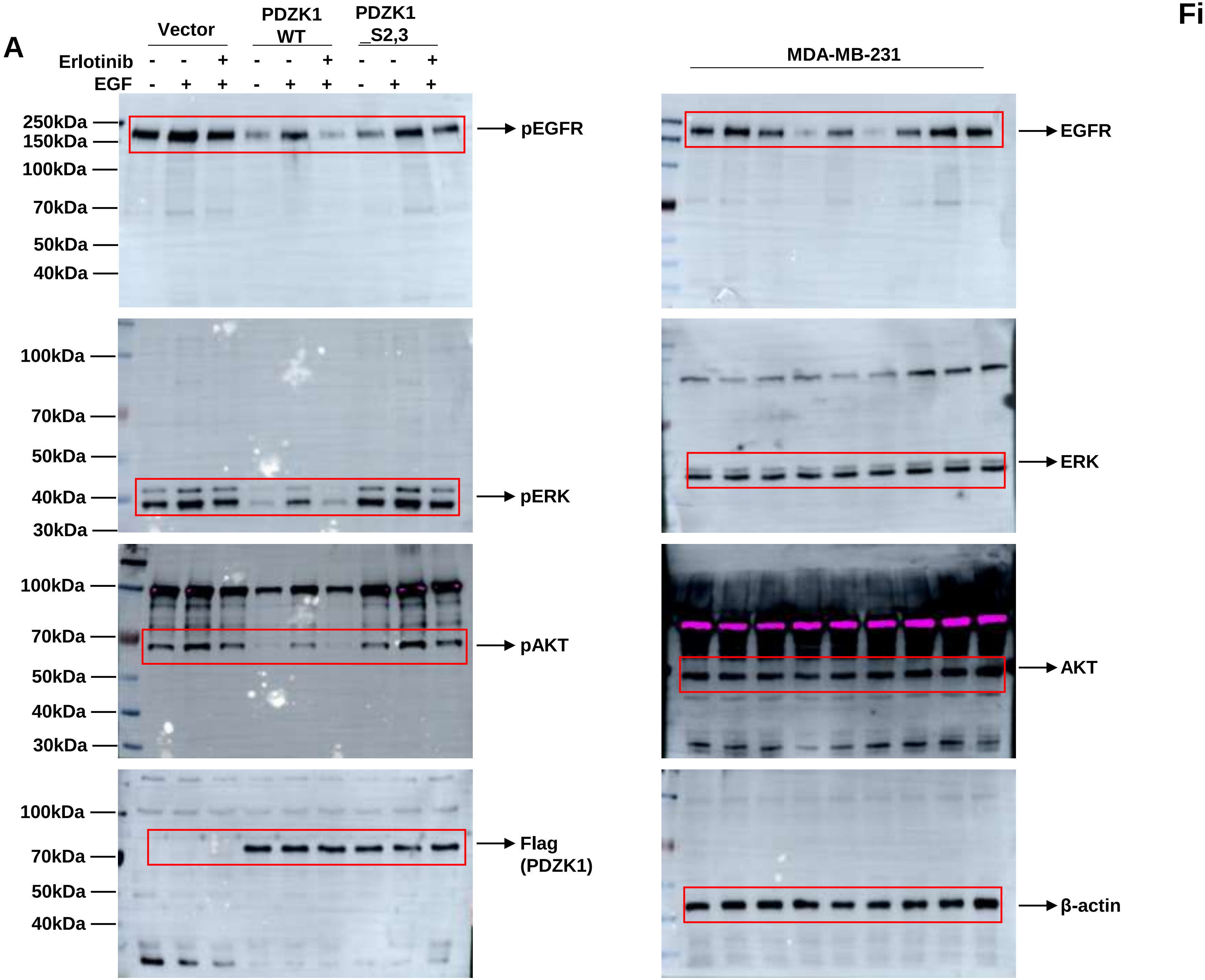
A
Vector
PDZK1 WT
PDZK1 _S2,3
Erlotinib - - + - - + - - +
EGF - + + - + + - + +
MDA-MB-231
250kDa
150kDa
100kDa
70kDa
50kDa
40kDa
30kDa
pEGFR
EGFR
pERK
ERK
pAKT
AKT
Flag (PDZK1)
β-actin

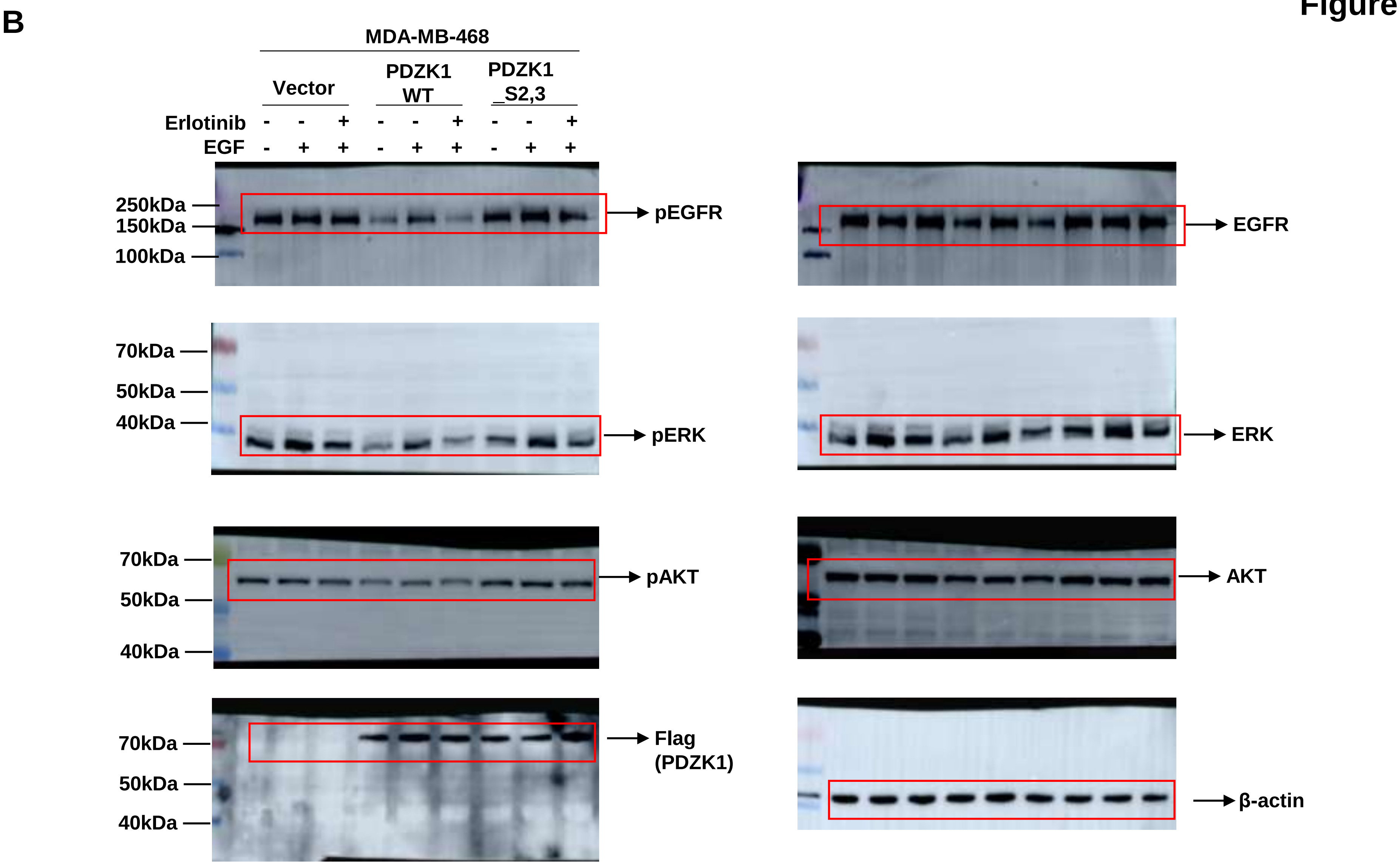
B
MDA-MB-468
Vector
PDZK1 WT
PDZK1 _S2,3
Erlotinib - - + - - + - - +
EGF - + + - + + - + +
250kDa
150kDa
100kDa
pEGFR
EGFR
70kDa
50kDa
40kDa
pERK
ERK
70kDa
50kDa
40kDa
pAKT
AKT
70kDa
50kDa
40kDa
Flag (PDZK1)
β-actin
